# The LUBAC subunit HOIL-1 promotes the progression of HBV-associated hepatocellular carcinoma independently of linear ubiquitination

**DOI:** 10.1038/s12276-025-01556-4

**Published:** 2025-10-06

**Authors:** Zheyu Dong, Qiuyue Ye, Yuxin Zhou, Yuqing Shao, Junling Chen, Jianzhong Cai, Yiyan Huang, Jiayue Yang, Yaoting Feng, Liangxing Chen, Libo Tang, Yuchuan Jiang, Peng Chen, Yu Wang, Yongyin Li

**Affiliations:** 1 State Key Laboratory of Organ Failure Research, Guangdong Provincial Key Laboratory of Viral Hepatitis Research, Department of Infectious Diseases, Nanfang Hospital, Southern Medical University, Guangzhou, China; 2 Department of Nephrology, Affiliated Hospital of Guizhou Medical University, Guiyang, China; 3 The First School of Clinical Medicine, Southern Medical University, Guangzhou, China; 4 Department of Gastroenterology, The Second Affiliated Hospital of Nanchang University, Nanchang, China; 5 Department of Hepatobiliary Surgery, Nanfang Hospital, Southern Medical University, Guangzhou, China

**Keywords:** Tumour virus infections, Liver cancer, Oncogenes

## Abstract

The linear ubiquitin chain assembly complex (LUBAC) has been implicated in both cancer progression and viral activity; however, its role in the progression of hepatitis B virus (HBV)-associated hepatocellular carcinoma (HBV-HCC) remains unclear. Here we found that the expression of LUBAC components and Met1-linked ubiquitination was significantly upregulated and associated with poor prognosis in HCC; however, blocking the LUBAC activity with HOIPIN-1 did not affect the malignancy of HCC cells or their sensitivity to sorafenib treatment. Targeting HOIL-1 inhibited the progression of HCC in vitro and in vivo. Interestingly, we found that *HOIL-1*, but not other LUBAC components, was exclusively upregulated in HBV-HCC. Functionally, HOIL-1 knockdown suppressed tumor growth, metastasis and stemness in HBV-infected HCC cells. Mechanistically, HOIL-1 interacted with HBx, but not other HBV proteins, and facilitated its stabilization by recruiting deubiquitinatinase USP15, thereby reducing HBx K48-linked ubiquitination. Notably, the clinical analysis indicated that the association between high HOIL-1 expression and poor prognosis was evident only in patients with HBV-HCC with high USP15 expression and not in those with low USP15 expression. Collectively, our results demonstrated that HOIL-1 acts as an oncogene to promote HBV-HCC progression independent of LUBAC activity and may serve as a potential therapeutic target for HBV-HCC.

## HOIL-1 drives HBV-HCC progression independent of LUBAC

Hepatocellular carcinoma (HCC) is a major cause of cancer deaths worldwide, often linked to hepatitis B virus (HBV) infection. Despite vaccines and treatments, HBV remains a key risk factor for HCC. This Article explores how HOIL-1 protein affects HCC progression in HBV-infected patients. Researchers found that HOIL-1, part of a protein complex called LUBAC, is active in HCC tissues. They studied human liver cancer cells and mice to understand HOIL-1’s role. They discovered that HOIL-1 helps stabilize another protein, HBx, which is crucial for HBV-related cancer development. HOIL-1 does this by interacting with an enzyme called USP15, preventing HBx from being broken down. The study shows that reducing HOIL-1 levels can slow down cancer growth and spread in HBV-related HCC. This suggests that targeting HOIL-1 could be a new way to treat this type of liver cancer.

This summary was initially drafted using artificial intelligence, then revised and fact-checked by the author.

## Introduction

Hepatocellular carcinoma (HCC) is the second-most common cause of cancer mortality worldwide^1^. Hepatitis B virus (HBV) is the leading cause of HCC^2^. Despite the prominent effect of the preventive vaccine for newborns and antiviral treatments, HBV remains the most important global risk factor of HCC, which is considered a challenging global public health issue. Previous studies have proved that the hepatitis B X protein (HBx) is essential for viral replication; in addition, it is also involved in regulating gene transcription, intracellular signal transduction, genotoxic stress response, protein degradation, cell cycle control and apoptosis and behaving as a prominent oncogenic driver for HBV-associated HCC (HBV-HCC)^2,3^. It has been demonstrated that various anti-HBV drugs can downregulate the expression of HBx, inhibiting the proliferation of HBV-HCC^4^. Of note, it also promotes the metastasis of HCC by regulating molecules associated with the migration and invasion of tumor cells^5,6^. Several studies on HBx transgenic mice have investigated the hepatocarcinogenic effects of HBx and found that only those with high HBx levels developed HCC^7,8^. HBx hijacking the ubiquitin–proteasome system was a central theme around virus-induced oncogenesis^9^. HBx is an unstable protein that is ubiquitinated and rapidly degraded by the proteasome pathway to maintain a very low intracellular level^10,11^. Clinical reports have presented HBx as a possible diagnostic marker by demonstrating its expression in 40% of sera and 85% of liver tissue samples from patients with HCC^12^. The versatile ubiquitin–proteasome system components, in turn, regulated the stability of HBx via direct or indirect mechanisms^13^. However, the underlying factors and mechanisms that maintain the stability of HBx for HBx-induced carcinogenesis are poorly understood.

Ubiquitination is an essential posttranslational modification that controls most cellular processes, including cell cycle progression, DNA damage response, gene transcription, receptor transport and protein stability^14^. Distinct from the biochemically interubiquitin linkages through the seven internal lysine residues of ubiquitin, the linear ubiquitin chain (also called Met1-linked polyubiquitination (M1-Ubi)) was recently found to assemble via peptide-bond formation between the α-amino group (α-NH_2_) of the N-terminal methionine (Met1) of one ubiquitin and the carboxyl group of the C-terminal glycine of another ubiquitin^15^. The linear ubiquitin chain assembly complex (LUBAC), the sole E3 ubiquitin ligase known to assemble linear ubiquitin chains specifically, is composed of hemoxidized IRP2 ubiquitin ligase 1L (HOIL-1; also known as RBCK1), HOIL-1-interacting protein (HOIP; also known as RNF31) and SHANK-associated RH-domain-interacting protein (SHARPIN)^16^. Among these three subunits of LUBAC, HOIP has a catalytic center in its RING2 domain responsible for assembling linear ubiquitin chains, whereas HOIL-1 and SHARPIN were recognized as accessory proteins for the process^15,17^. HOIL-1 and SHARPIN harbor ubiquitin-like domains that interact with the ubiquitin-binding domains in HOIP to form a stable complex, thereby exhibiting ubiquitin ligase activity as involved in biological functions including immune signaling, development in mice, protein quality control, Wnt signaling and autophagy^18^. Increasing evidence shows that the components of LUBAC and it-mediated M1-Ubi are linked to cancer progression; however, the underlying mechanism for regulating HBV-HCC remains largely unknown. Our previous study indicated that HOIL-1 played a critical role in regulating the sorafenib resistance and cancer stemness of HCC, which was independent of the LUBAC activity^19^.

In this Article, we found that the expression of LUBAC and M1-Ubi was upregulated in HCC; however, LUBAC promoted the progression of HCC independent of its M1-Ubi activity. Interestingly, only HOIL-1 was overexpressed and correlated with tumor malignancy and poor prognosis of patients with HBV-HCC. Knocking down HOIL-1 significantly impaired the HBV-HCC cell growth, migration and cancer stem cell (CSC) properties in vitro and inhibited tumor progression in an HBV-HCC mouse model. Mechanistically, HOIL-1 interacted with HBx and recruited deubiquitylating enzyme USP15 to promote its stabilization by decreasing the K48-linked ubiquitination modification. Thus, our study suggests that HOIL-1 mediating HBx stability is critical in the process of HBV-HCC development, which may be a novel target for HCC therapeutics.

## Materials and methods

### HCC cell lines

The human HCC cell lines Huh7 (Research Resource Identifier (RRID): CVCL_0336), PLC/PRF5 (RRID: CVCL_0485), HCCLM3 (RRID: CVCL_6832), MHCC97H (RRID: CVCL_4972), HepG2.2.15 (RRID: CVCL_L855) and HepG2 (RRID: CVCL_0027) were all obtained from Shanghai Institute for Biological Sciences. All cell lines have been authenticated within the last 3 years via short tandem repeat analysis. Experiments were performed in mycoplasma-free cells. The cells were routinely cultured in the Dulbecco’s modified Eagle medium containing 10% fetal bovine serum (ExCell) at 37 °C in a humidified atmosphere of 5% CO_2_.

### HCC tissue specimens

The current study was conducted on 147 paraffin-embedded, archived HCC specimens, which were histopathologically and clinically diagnosed at the Nanfang Hospital from 2015 to 2016 (Supplementary Table 1). The samples were selected on the basis of specific inclusion criteria: all patients were clinically diagnosed with HCC for the first time, and they did not receive any chemotherapy, radiotherapy or surgical treatment; the general condition of patients was good, and the clinical data were complete. The exclusion criteria were as follows: condition combined with other types and sites of malignant tumors or the presence of systemic HCC metastasis. HCC tissues and paired adjacent nontumor tissues were frozen in liquid nitrogen until further use. Adjacent nontumor tissues were obtained from a standard distance (3 cm) from resected neoplastic tissues of patients with HCC who underwent surgical resection and were confirmed by pathological evaluation. The prior patient consent and approval from the Institutional Research Ethics Committee of Nanfang Hospital were obtained for these clinical materials for research purposes. It also conformed to the provisions of the Declaration of Helsinki.

### Animals

C57BL/6 and BALB/c nude mice were purchased from the Laboratory Animal Center of Southern Medical University. Mice were housed in a temperature-controlled animal facility (20–22 °C) under 12–12 h light–dark cycle. The mice had free access to water and food. All animal work was approved by the Ethical Committee for Experimental Animal Care of Southern Medical University (Guangzhou, China). The studies reported in this paper are in accordance with the Animal Research: Reporting of In Vivo Experiments guidelines.

#### HBV mouse model and antigen detection

The 6- to 8-week-old male C57BL/6 mice were used to construct the HBV mouse model. The mice were hydrodynamically injected via the tail vein with 10 µg of the pAAV-HBV1.2 plasmid (genotype A), diluted in normal saline to a volume corresponding to 10% of the mouse’s body weight. The serum samples were collected at specified time points for HBsAg and HBeAg detection using the Roche COBAS 6000 analyzer (Roche Molecular Diagnostics) to assess whether the model was successfully established.

#### DEN/CCl_4_-induced HCC mouse model

The mice were injected with a single dose of diethylnitrosamine (DEN) (25 mg/kg) by intraperitoneal injection at the age of 14 days. At 8 weeks, all mice were subjected to CCl_4_ (0.2 ml/kg, intraperitoneal) once a week for up to 14 consecutive weeks. For the intrahepatic knockdown of HOIL-1, the custom-made adenoviral vector carrying shRNA for mouse HOIL-1 (AAV9-shHOIL-1, shRNA sequence: AGACGACAGCGATGCTAAA) or control were purchased from Hanbio Biotechnology. The adenoviral was injected into the mice (1 × 10^11^ plaque-forming units per mouse, via tail vein) after the 10-week injection of CCl_4_ (18-week age). All the mice were killed after the 4-week injection of adeno-associated virus serotype 9 (AAV9).

#### Subcutaneous implantation HCC mouse models

The 4-week-old male BALB/c nude mice (Hua Fukang Bio) received subcutaneous injections of 5 × 10^6^ HCCLM3 cells overexpressed with LV-HOIL-1 or LV-Ctrl or Huh7 cells transduced with shHOIL-1 or shCtrl. Tumor growth was monitored every week using calipers. Tumor volume was calculated using the formula: *V* = (length × width²)/2. All mice were killed after 4 weeks.

### In vitro regulating the expression of HOIL-1

For the silencing of HOIL-1 in HCC cells, shRNA sequences against human HOIL-1 (shHOIL-1: GGTGCACCTTCATCAACAA) (GeneChem) were transduced into HepG2.2.15 cells. The transduced cells were selected with 5 µg/ml puromycin.

### Cell viability assay

The cell viability was evaluated using a cell counting kit 8 (CCK-8) (B34302, Bimake) according to the manufacturer’s instructions. In brief, the indicated cells (4 × 10^3^ per well) were inoculated in 96-well plates for 24, 48, 72 and 96 h, respectively. The CCK-8 solution (10 μl) was added to each well, and the plates were incubated for 2 h. The absorbance at 450 nm was then analyzed and calculated.

### Transwell migration assay

The Transwell migration assay was performed using 8-μm-pore polycarbonate membrane inserts according to the manufacturer’s instructions. In brief, cells were resuspended by fetal bovine serum-free starvation medium and then planted in the inserts, and growth medium was added outside the chamber in the wells of the plate. After 12 h of incubation, the cells that got through the compartment were stained using Hoechst 33342 (Thermo Scientific). Then, the stained cells were counted as the mean number of cells per field of view.

### Cell cycle analysis

The cell cycle of the indicated cells was determined using the cell cycle detection Kit (BD) according to the manufacturer’s instructions by a fluorescence-activated cell sorting Calibur machine using CellQuest software (BD Biosciences). The data were analyzed using FlowJo software.

### RNA extraction and qRT–PCR

The quantitative real-time polymerase chain reaction (qRT–PCR) was performed as described previously^20^. The primer sequences used for HOIL-1 were: forward: 5′-TGCTCAGATGCACACCGTC-3′; reverse: 5′-CAAGACTGGTGGGAAGCCATA-3′. For glyceraldehyde 3-phosphate dehydrogenase (GAPDH), the sequences used were: forward: 5′-TGCACCACCAACTGCTTAGC-3′； reverse: 5′-GGCATGGACTGTGGTCATGAG-3′. GAPDH was used as an internal control.

### Western blot analysis

The western blot analysis was performed as described previously^20^. The anti-GAPDH antibody was used for the normalization of protein expression. Bands were detected using an ECL Detection Kit (Millipore) and visualized using the enhanced chemiluminescent imaging system (Sage Creation) according to the manufacturer’s instructions.

### Immunoprecipitation

In brief, cells were collected and lysed for 30 min on ice and centrifuged at 12,000 rpm for 15 min. Soluble lysates were incubated with the indicated antibodies overnight at 4 °C, followed by incubation with protein A/G magnetic beads (Bimake) at 4 °C for another 2 h. The immunocomplex was washed five times with cold immunoprecipitation wash buffer (150 mM NaCl, 10 mM HEPES pH 7.4, 0.1% NP-40) and boiled in 1× sodium dodecyl sulfate sample buffer for 5 min. The coprecipitate was resolved using sodium dodecyl sulfate–polyacrylamide gel electrophoresis and immunoblotted with specific antibodies. The protein bands were visualized using the enhanced chemiluminescent imaging system (Sage Creation) according to the manufacturer’s instructions.

### ChIP

Chromatin immunoprecipitation (ChIP) was performed using a ChIP assay kit (Cell signal technology, no. 9003) according to the manufacturer’s protocol. In brief, 1 × 10^7^ HepG2.2.15 cells were treated as indicated for 24 h, and the were crosslinked with 1% formaldehyde at room temperature for 10 min and quenched with 125 mM glycine. The cells were then lysed, and the chromatin was sonicated to an average fragment size of 200–500 bp. The sheared chromatin was incubated overnight at 4 °C with specific antibodies against p65 or control IgG. Antibody-bound complexes were pulled down using protein A/G magnetic beads, followed by a series of wash steps. Crosslinks were reversed by heating at 65 °C overnight, and DNA was purified using spin columns. The ChIP-enriched DNA was subjected to PCR or real-time PCR using the primer: MCP-1 promoter containing the p65 binding site (forward; 5′-TGTAATTCCACCAGAGTCTGAAA-3′ and reverse: 5′-TCCATTCCAGAATCTCTTCTTCCT-3′). Data were normalized to input DNA and presented as fold enrichment relative to IgG controls.

### IHC

Human HCC surgical specimens were fixed in 10% neutral formalin-fixed, dehydrated and embedded in paraffin. Tissues were cut into 4-μm-thick sections and incubated in citrate buffer (pH 9.0) for 5 min at 120 °C; then, the endogenous peroxidase was blocked by 3% H_2_O_2_ for 10 min at room temperature. The slides were incubated with 10% normal goat serum in PBS for 30 min at 37 °C to block the nonspecific binding sites, followed by incubation with the appropriate primary antibodies overnight at 4 °C. Then, the the streptavidin-peroxidase kit (ZSGB-Bio) was used according to the manufacturer’s instructions. The target protein expression levels were independently evaluated by two pathologists.

### GSEA and public data deposition

For gene set enrichment analysis (GSEA), we obtained the GSEA software (version 4.03) from the website (http://software.broadinstitute.org/gsea/index.jsp). The data were downloaded from the The Cancer Genome Atlas (TCGA) liver HCC (LIHC) cohort and GSE14520 cohort. The samples in the TCGA cohort were divided into two groups according to the expression of HOIL-1. Then, we obtained the GMT files (c2.all.v7.2.symbols.gmt (Curated)) from the molecular signatures database (http://www.gsea-msigdb.org/gsea/downloads.jsp). Based on the gene expression profile and phenotype grouping, the minimum gene set was ten. The maximum gene set was 500, with 1000 times resampling; a *P*_adj_ value (adjust method: Benjamini–Hochberg) <0.05 and a false discovery rate <0.25 were considered statistically significant.

### Transcriptomic analysis

The RNA extracted from HCCLM3 cells transduced with HOIL-1, Huh7 cells transduced with shHOIL-1 and their corresponding control cells. RNA extraction and the measurement of RNA quality and quantity were conducted as previously described^19^. The RNA sequencing coverage and quality statistics were presented in Supplementary Table 2.

### Statistical analysis

Data were expressed as the mean ± s.d. The student’s *t-*test or the Mann–Whitney *U* test was used when two groups were compared. The Kruskal–Wallis *H* test or one-way analysis of variance was used to compare more than two groups. Correlations between variables were assessed with Spearman’s rank order correlation coefficient. Survival curves were analyzed by the Kaplan–Meier method, and a log-rank test was used to assess significance. Asterisk coding as indicated in the figure legends as *P < 0.05; **P < 0.01; ***P < 0.001, ****P < 0.0001. The patients were divided into two groups according to whether they exhibited a high (greater than the median) or low (less than the median) HOIL-1 expression level. Statistical parameters, including the number of biological replicates and repeat experiments, are reported in the figure legends. All calculations were performed using GraphPad prism 9.0 or the SPSS software package.

## Results

### Pharmacological targeting of LUBAC does not exert significant inhibitory effects on HCC cell proliferation in vitro

**Fig. 1:**
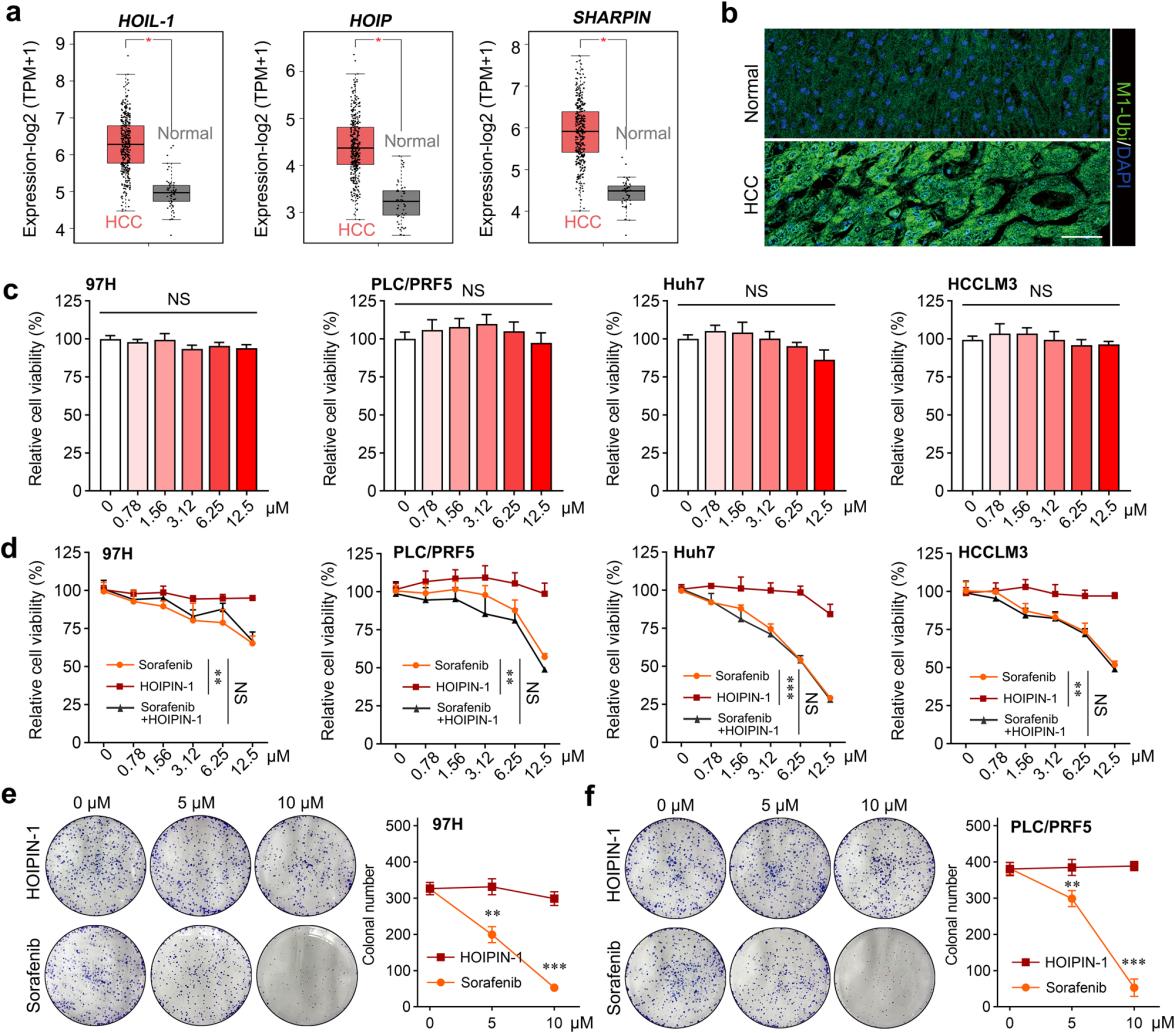
Targeting LUBAC does not exert significant inhibitory effects on HCC cell proliferation in vitro. **a** The mRNA levels of *HOIL-1*, *HOIP* and *SHARPIN* in the TCGA LIHC cohort. **b** The immunofluorescence staining of M1-Ubi in HCC and normal samples. Scale bar, 20 μm. **c** The cell viability of HCC cells treated with different concentrations of HOIPIN-1 for 48 h. **d** The cell viability of HCC cells treated with different concentrations of HOIPIN-1/sorafenib/HOIPIN-1+sorafenib for 48 h. **e**, **f** The colony-forming capacity of 97H (**e**) and PLC/PRF5 (**f**) cells treated with different concentrations of HOIPIN-1/sorafenib for 48 h and the quantification of the colony formation. The Mann–Whitney *U* test was used in **a**, **d**, **e** and **f** and the Kruskal–Wallis *H* test in **c**. Data are shown as mean ± s.d., *P < 0.05, **P < 0.01, ***P < 0.001. NS, not significant; DAPI, 4′,6-diamidino-2-phenylindole.

To investigate the role of LUBAC in HCC, we first detected the expression of LUBAC components and M1-Ubi in HCC tissues. As indicated in Fig. 1a, we found that the expression of LUBAC components was significantly upregulated in HCC tissues compared with the normal tissues, as was the expression of M1-Ubi (Fig. 1b). Furthermore, the high expression of LUBAC components was associated with poor prognosis in HCC (Supplementary Fig. 1a–c). HOIPIN-1 is reported as a LUBAC inhibitor, inhibiting the in vitro linear ubiquitination activity by the truncated LUBAC at an IC_50_ value of 2.8 μM (ref. ^21^). However, the HOIPIN-1 treatment, which was sufficient to inhibit the formation of linear ubiquitin chains, NFκB signaling activation and the M1 ubiquitin of IKBKG at applied dosage (Supplementary Fig. 1d,e), had no significant inhibitory effect on the proliferative and clonal ability of HCC cells (Fig. 1c–f). On the contrary, sorafenib, the first-line treatment for advanced HCC, exerted a significant inhibitory effect on the proliferation and clonogenic potential of HCC cells in a concentration-dependent manner and also showed a more potent antitumor activity compared with HOIPIN-1 at the same concentration (Fig. 1d–f). In addition, cotreatment with HOIPIN-1 did not significantly enhance the growth-inhibitory effect of sorafenib on HCC cells compared with sorafenib alone (Fig. 1d). Thus, these results indicate that LUBAC may promote the progression of HCC independent of its LUBAC activity, and targeting LUBAC activity with inhibitors may not be an optimal strategy for HCC treatment.

### The LUBAC component HOIL-1 is associated with tumor progression and poor prognosis in HCC

**Fig. 2:**
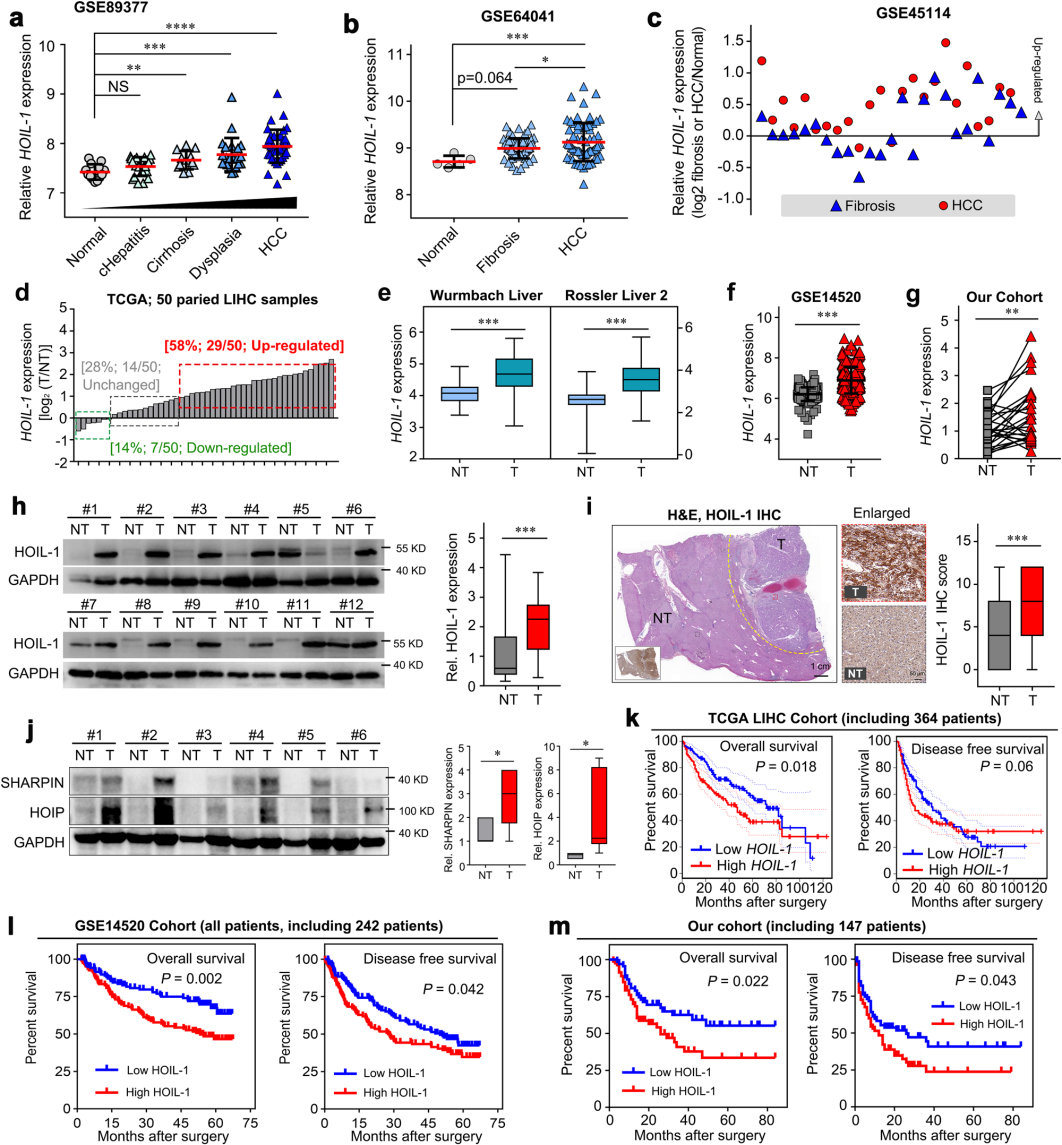
Upregulated HOIL-1 expression associated with tumor progression in HCC. **a** The expression of HOIL-1 in different stages of HCC development (including normal, chronic hepatitis, cirrhosis, dysplasia and HCC) in the GSE89377 cohort. **b** The expression of HOIL-1 in different stages of HCC development (including normal, fibrosis and HCC) in the GSE64041 cohort. **c**, The expression of HOIL-1 in fibrosis and HCC tissues relative to the normal tissues in the GSE45114 cohort. **d**–**g** The mRNA expression of *HOIL-1* in tumor and nontumor tissues in TCGA LIHC (**d**), Oncomine (**e**), GSE14520 (**f**) and our (**g**) cohorts. **h**, **i** The expression of HOIL-1 in tumor and nontumor tissues was detected by western blot (h) and IHC (i) assays. **j** The expression of HOIP and SHARPIN in tumor and nontumor tissues was detected by western blot and the relative quantification. **k** The Kaplan–Meier analysis for OS and disease-free survival of patients with HCC with low and high expression of HOIL-1 in the TCGA LIHC cohort. **l**, **m** The Kaplan–Meier analysis for OS and disease-free survival of patients with HCC with low and high expression of HOIL-1 in GSE14520 (**l**, including all patients) and our cohort (**m**). The Kruskal–Wallis *H* test was used in **a** and **b**, the Mann–Whitney *U* test in **e**–**j** and the log-rank Mantel–Cox test in **k** and **l**. Data are shown as mean ± s.d., *P < 0.05, **P < 0.01, ***P < 0.001, ****P < 0.0001. NS not significant, H&E hematoxylin and eosin staining, NT nontumor, T tumor.

The published studies and our previous study have indicated that the LUBAC components promote the progression of tumors independent of LUBAC activity via regulating the activation of oncogenic pathways^19,22,23^. Our previous study indicated that the LUBAC component HOIL-1 played a critical role in regulating the sorafenib resistance and cancer stemness of HCC and was independent of the LUBAC activity^19^. Thus, in the present Article, we sought to investigate the role of HOIL-1 in regulating the growth of HCC and to explore the association of HOIL-1 expression with HCC prognosis. Interestingly, we found that HOIL-1 expression was gradually increased in the different stages of HCC and significantly upregulated in HCC compared with that in the other stages (Fig. 2a–c), indicating a key role of HOIL-1 in the tumorigenesis of HCC. In addition, data from the TCGA LIHC dataset indicated that *HOIL-1* mRNA levels were upregulated in 58% of HCC cases (29/50; log_2_(fold change) >1; Fig. 2d) and significantly elevated in the tumor tissues when compared with that in nontumor tissues in the Oncomine and GEO cohorts (Fig. 2e, f). Similarly, RT–qPCR results revealed a significant increase in *HOIL-1* mRNA expression in tumor tissues than in the paired nontumor tissues in our cohort (Fig. 2g). Consistently, both western blot and immunohistochemistry (IHC) analyses demonstrated elevated HOIL-1 protein levels in samples (Fig. 2h, i). In addition, the protein levels of the other two components of LUBAC, HOIP and SHARPIN were upregulated in the tumor tissues, as evidenced by western blot (Fig. 2j). Notably, the Kaplan–Meier curves analysis demonstrated that the increased expression of HOIL-1 was associated with the poor prognosis of patients with HCC in both the GEO, TCGA and our cohort (Fig. 2k–m). Furthermore, univariate and multivariate regression analyses revealed that increased HOIL-1 expression was an independent prognostic factor associated with shorter overall survival (OS) (Supplementary Fig. 2a, b and Supplementary Table 1). Collectively, HOIL-1, as a key component of LUBAC, plays an important role in the tumorigenesis and malignant properties of HCC.

### Targeting HOIL-1 inhibits the progression of HCC by regulating the oncogenic pathways

**Fig. 3:**
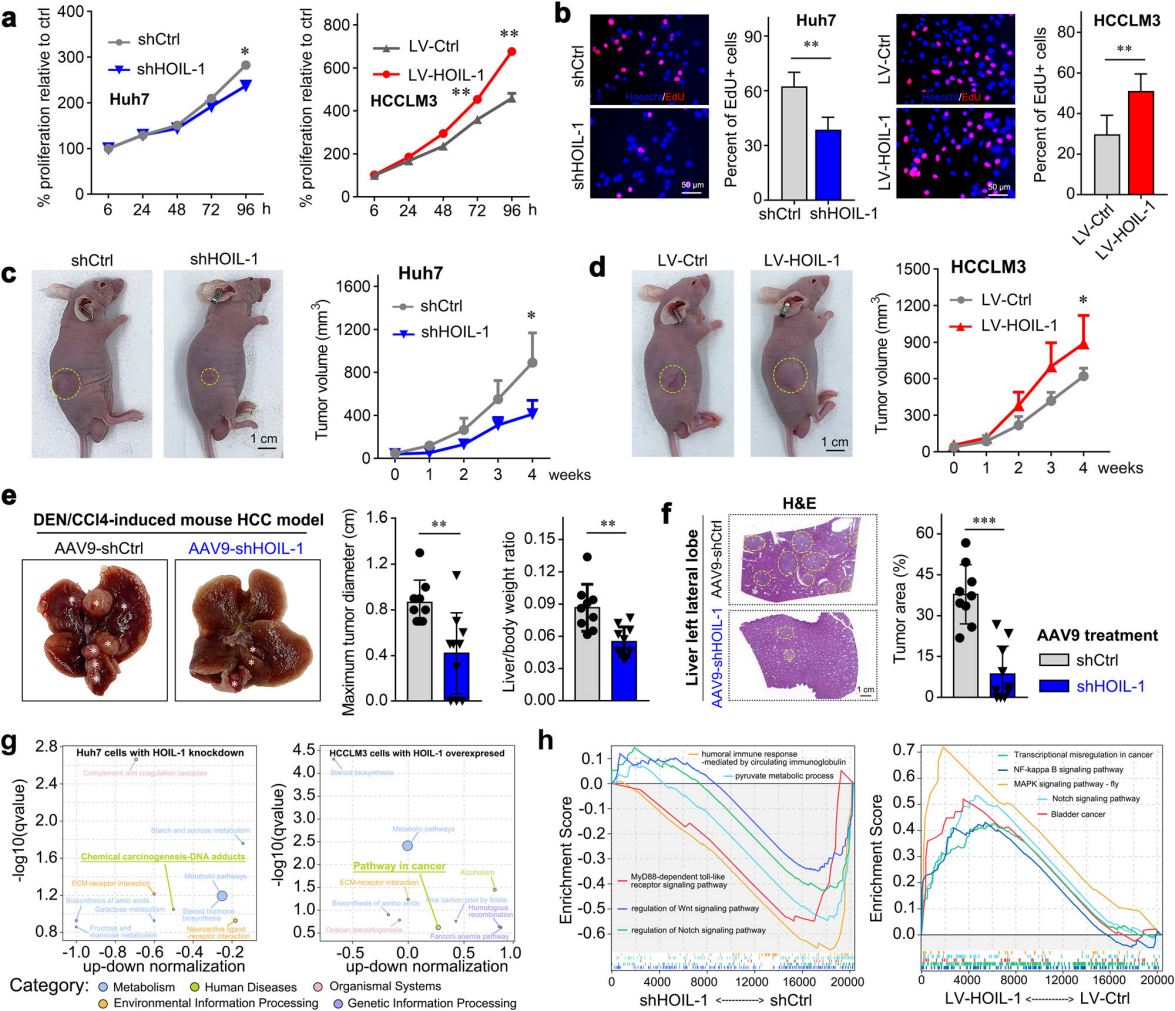
Targeting HOIL-1 expression inhibits the growth of HCC by regulating oncogenic pathways. **a** The proliferation of Huh7 and HCCLM3 cells with the indicated treatment. **b** The EdU incorporation assays of Huh7 and HCCLM3 cells with the indicated treatment. **c**, **d** The representative image of the mouse bearing Huh7 (**c**)/HCCLM3 (**d**) cells and the quantification of tumor growth, *n* = 6. **e** The representative liver image of the DEN/CCl_4_-induced mouse HCC model, the quantification of maximum tumor diameter and the liver/body weight ratio (*n* = 9). **f** The representative image of hematoxylin and eosin (H&E) staining and the quantification of the tumor area. **g** The KEGG analysis of the differentially expressed genes in Huh7 and HCCLM3, treated as indicated. **h** The GSEA of Huh7 and HCCLM3, treated as indicated. The Mann–Whitney *U* test was used in **a**–**f**. Data are shown as mean ± s.d., *P < 0.05, **P < 0.01, ***P < 0.001. LV, lentiviral; sh, small hairpin.

To verify the role of HOIL-1 in regulating the growth of HCC, we performed in vitro and in vivo studies. Primary, we detected the HOIL-1 expression in HCC cell lines (including Huh7, HepG2, MHCC97H, PLC/PRF5 and HCCLM3) and normal hepatocyte MIHA. We found that Huh7 cells exhibit relatively high endogenous HOIL-1 expression, making them more suitable for the knockdown experiments, whereas HCCLM3 cells have lower HOIL-1 levels, allowing the clearer interpretation of overexpression effects (Supplementary Fig. 3a). We manipulated the expression of HOIL-1 in HCC cells using the lentivirus (Supplementary Fig. 3b–e) and found that HOIL-1 knockdown impaired the proliferative capacity of HCC cells, whereas HOIL-1 overexpression had an opposite effect (Fig. 3a, b and Supplementary Fig. 3f). Consistently, we found that the knockdown of the LUBAC component HOIP or SHARPIN impaired complex formation, as indicated by the reduced levels of M1-Ubi, and suppressed the proliferation of HCC cells but did not change HOIL-1 expression (Supplementary Fig. 4a–e). More importantly, under the condition of HOIP or SHARPIN knockdown, HOIL-1 overexpression was still sufficient to promote HCC cell proliferation (Supplementary Fig. 4f,g), suggesting a LUBAC-independent oncogenic role of HOIL-1 in HCC progression.

Next, to confirm the oncogenic role of HOIL-1 in vivo, we applied subcutaneous HCC nude mouse models by subcutaneously injecting 5 × 10^6^ Huh7 (transfected with shHOIL-1 or shCtrl) or HCCLM3 (transfected with LV-HOIL-1 or LV-Ctrl) cells. As shown in Fig. 3c, HOIL-1 knockdown significantly inhibited the growth of the tumor model bearing Huh7 cells. The tumors with HOIL-1 overexpression showed a faster growth rate (Fig. 3d). Owning to the lack of liver microenvironment in the subcutaneous implantation HCC mouse model, we further established the DEN/CCl_4_-induced HCC mouse models, which could more closely mimic the development of human HCC. The DEN/CCl_4_-induced HCC mouse models were intravenously injected with AAV9 carrying shCtrl or shHOIL-1. Previous studies have shown that adeno-associated virus (AAV) activates interferon (IFN)-Ⅰ signaling and interferon-stimulated gene (ISG) responses, which exert antitumor or protumor effects^24,25^. To determine whether the antitumor effects are due to HOIL-1 knockdown rather than AAV-induced immune responses, we treated DEN/CCl_4_-induced HCC mouse models with or without AAV9-shCtrl. Although AAV9-mediated activation of IFN-Ⅰ and ISG responses were observed (Supplementary Fig. 5a,b), indicated by the expression of p-STAT1 and ISGs (OAS1B and SOCS1), there were no differences in the maximum tumor diameter and liver/body weight ratio between the two groups (Supplementary Fig. 5c). In the AAV9-shHOIL-1-treated mice, we observed a significant knockdown of the HOIL-1 expression in tumor tissues (Supplementary Fig. 5d,e) and alleviated tumor progression compared with control treatment, as evidenced by an evident reduction in the maximum tumor diameter, liver/body ratio and tumor area (Fig. 3e, f).

To explore the mechanism with which HOIL-1 regulates the malignancy of HCC, we subjected the HCC cells transduced with shHOIL-1 or LV-HOIL-1 and their control cells to RNA sequencing. A total of 572 differentially expressed genes in Huh7 cells transduced with shHOIL-1 (with a false discovery rate value <0.05 and a fold change >|2|) were identified as well as 2,036 in HCCLM3 cells transduced with LV-HOIL-1 (Supplementary Fig. 6a–c). Notably, RT–qPCR validation confirmed that over 80% of the top ten differentially expressed genes exhibited consistent changes with the RNA sequencing data in both Huh7 and HCCLM3 cells (Supplementary Fig. 6d,e). By the Kyoto Encyclopedia of Genes and Genomes (KEGG) pathway enrichment analysis, these genes were categorized into metabolism, biosynthesis, extracellular matrix (ECM)-receptor and tumor-associated pathways (Fig. 3g). The GSEA indicated that the HOIL-1 expression was associated with the activation of the metabolic, WNT and Notch pathways, which is consistent with the previous studies (Fig. 3h). Taken together, HOIL-1 promotes HCC progression by regulating the well-established oncogenic pathways.

### The LUBAC component HOIL-1, not HOIP or SHARPIN, is upregulated in HBV-HCC and associated with poor prognosis

**Fig. 4:**
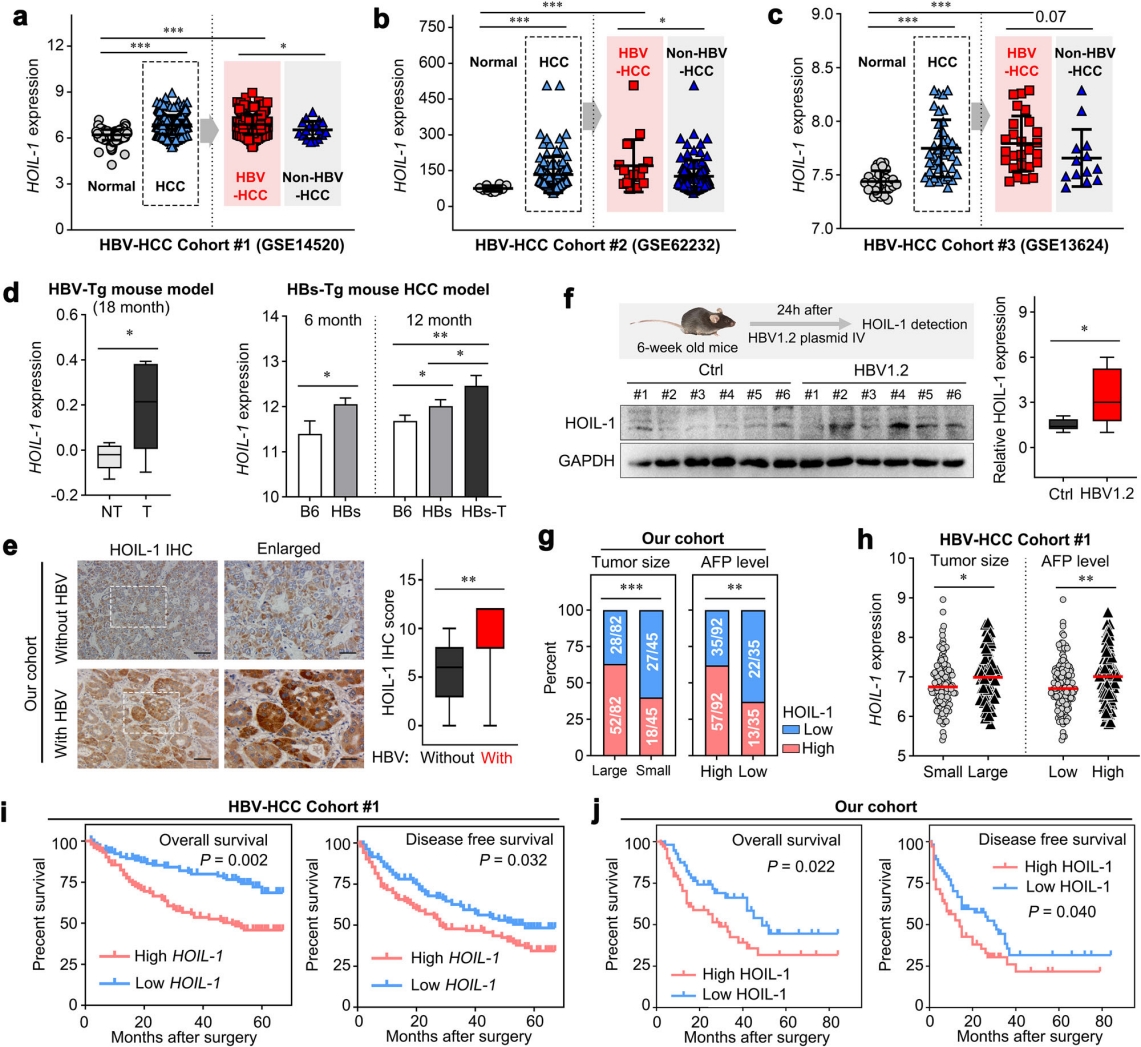
HOIL-1 expression is upregulated and associated with poor prognosis in HBV-HCC. **a**–**c** The expression of *HOIL-1* in the GSE14520 (**a**), GSE62232 (**b**) and GSE136247 (**c**) cohorts. **d** The expression of HOIL-1 in tumor and nontumor tissues in HBV-transgenic (HBV-Tg) HCC mouse models in the GSE103205 (left) cohort and hepatitis B surface antigen-transgenic (HBs-Tg) HCC mouse models in the GSE130668 (right) cohort. **e** The IHC detection of the HOIL-1 expression in HBV- and non-HBV-HCC tissues and the quantification of the IHC score. **f** The expression of HOIL-1 of HBV mouse model by hydrodynamic injection with the pAAV-HBV1.2 plasmid. **g**, **h** The expression of HOIL-1 in large (diameter >5 cm) and small (diameter ≤5 cm) tumor tissues (**g**) and in tumor tissues from patients with HCC with high (>300 ng/ml) and low (≤300 ng/ml) AFP levels (**h**) in the GSE14520 cohort and our cohort. **i**, **j** The Kaplan–Meier analysis for the OS and DFS of two groups of HBV-infected HCC patients defined by low and high expression of HOIL-1 in GSE14520 (**i,** only including 211 HBV-infected patients) and our cohort (**j**, only including 127 HBV-infected patients). The Kruskal–Wallis *H* test was used in **a**–**d**, the Mann–Whitney *U* test in **e**–**g** and the log-rank Mantel–Cox test in **h**. Data are shown as mean ± s.d., *P < 0.05, **P < 0.01, ***P < 0.001. DFS disease-free survival, IV intravenous.

As chronic HBV infection is one of the most important global risk factors of HCC, we sought to investigate the expression of LUBAC components associated with HBV infection in HCC. We first detected the expression of LUBAC components in HBV-HCC public datasets. Accordingly, we found that the expression of LUBAC components was significantly upregulated in HCC tissues compared with that in normal tissues (Fig. 4a–c and Supplementary Fig. 7a–c). Interestingly, subgroup analysis revealed that only *HOIL-1*, not *HOIP*/*SHARPIN*, showed an elevated level in HBV-HCC tissues compared with that in non-HBV-HCC tissues (Fig. 4a–c and Supplementary Fig. 7a–c). Similarly, we noticed an upregulated *HOIL-1* expression in the tumor tissues of the HBV-induced mouse HCC model (Fig. 4d). In our HBV-HCC cohort, we found HOIL-1 was upregulated in HBV-HCC compared with that in non-HBV-HCC (Fig. 4e). Moreover, to further explore the regulation of HBV on HOIL-1 expression, we constructed an HBV mouse model by hydrodynamic injection with the pAAV-HBV1.2 plasmid^26^, and the serum HBsAg and HBeAg were elevated at the 24-h time point (Supplementary Fig. 8a,b). In the HBV model, we found that the protein levels of HOIL-1 were elevated in the liver, the natural target organ of HBV infection (Fig. 4f), whereas the HOIP and SHARPIN showed no difference compared with control mice (Supplementary Fig. 8c). More interestingly, we found that HOIL-1 expression was higher in the livers of patients with HBV and those with elevated alanine aminotransferase or aspartate aminotransferase levels compared with their control (Supplementary Fig. 9a,b); however, HBV infection did not alter the HOIL-1 expression in HCC cells compared with their parental counterparts nor did the transfection with plasmids encoding HBV antigens affect the HOIL-1 levels in vitro (Supplementary Fig. 9c,d). Furthermore, the serial of inflammatory cytokines treatment showed that IFN-γ upregulated HOIL-1 expression (Supplementary Fig. 9e), which indicated that HOIL-1 upregulation may be at least partly mediated by HBV-induced cytokine responses rather than a direct viral effect.

To figure out the relationship between the expression of LUBAC components and tumor malignancy or patients’ prognosis, the datasets of chronic HBV carriers from GSE14520 and our cohort were applied for survival analysis. As indicated in Fig. 4g, h, we found that patients with higher *HOIL-1* expression showed larger tumor size and elevated α-fetoprotein (AFP) compared with the control, which was not observed regarding *SHARPIN* and *HOIP* expression (Supplementary Fig. 10a). As for the survival analysis, we found that patients with high *HOIL-1* but not *HOIP*/*SHARPIN* expression predicted poor prognosis (Fig. 4i, j and Supplementary Fig. 10b,c). In addition, univariate and multivariate Cox regression analysis revealed that HOIL-1 expression was an independent risk factor for patients with HCC, regardless of AFP levels, liver cirrhosis, tumor size and other clinical variables (Supplementary Fig. 10d,e). Thus, these results indicate that HBV infection exerts profound effects on LUBAC via regulating the expression of HOIL-1 but not HOIP or SHARPIN, suggesting that HOIL-1 is the critical component of LUBAC playing an important role in HBV-HCC.

### Targeting HOIL-1 significantly inhibits the malignant behavior of HBV-HCC in vitro and in vivo

**Fig. 5:**
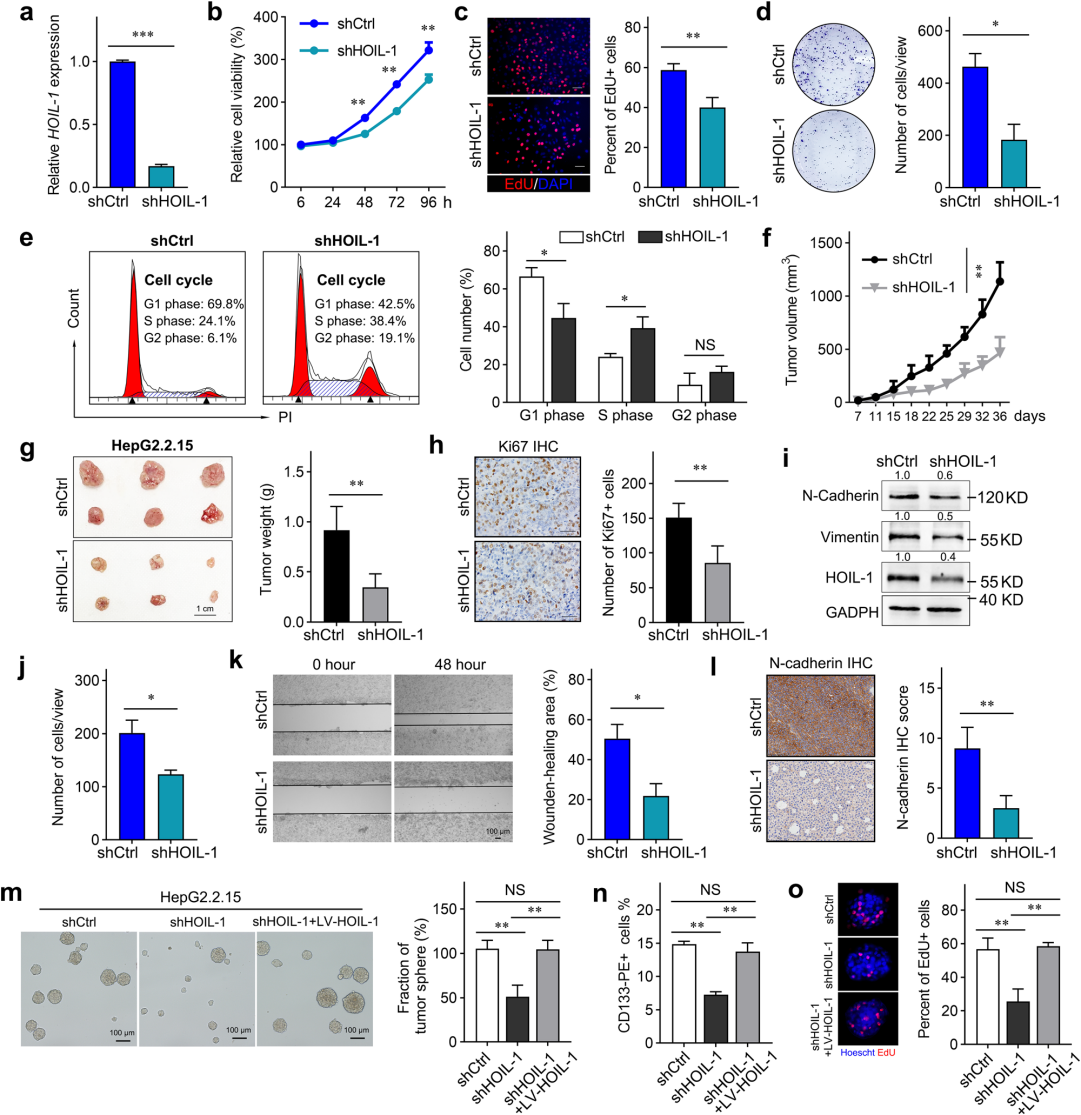
Knocking down the expression of HOIL-1 inhibits the progression of HBV-HCC in vitro and in vivo. **a** The qRT–PCR analysis of *HOIL-1* mRNA expression in HepG2.2.15 cells infected with the HOIL-1-knockdown lentivirus or the control lentivirus. **b** The effect of HOIL-1 knockdown on cell proliferation was detected by CCK-8. **c** The effect of HOIL-1 knockdown on cell proliferation was detected by EdU incorporation. **d** The effect of HOIL-1 knockdown on cell proliferation was detected by colony formation assays. **e** The distribution of different cell cycle phases in the HOIL-1-knockdown HepG2.2.15 cells. **f** The subcutaneous xenograft tumors of the indicated cells, *n* = 6; tumor volumes were measured and recorded twice a week, and a growth curve was plotted. **g** The representative image of indicated xenograft tumors and the quantification of the wet weight of tumors. **h** The representative images and quantification of IHC staining of Ki-67. **i** A western blot of the mesenchymal marker expression. **j**, **k** The migration capacities indicated by Transwell (**j**) and wound-healing (**k**) assays in HepG2.2.15 cells infected with the HOIL-1-knockdown lentivirus or the control lentivirus. **l** The representative images and quantification of IHC staining of N-cadherin in the xenograft tumors. Scale bars, 100 μm. **m**–**o** The tumorsphere formation assays (**m**), CD133^+^ population (**n**), EdU incorporation (**o**) of HepG2.2.15 transduced with shHOIL-1 or shCtrl, or LV-HOIL-1. The Mann–Whitney *U* test was used in **a**–**e** and **g**–**i**. Data are shown as mean ± s.d., *P < 0.05, **P < 0.01, ***P < 0.001.

To elucidate the functional role of HOIL-1 in HBV-HCC, we stably knocked down HOIL-1 expression in HepG2.2.15 cells with lentivirus (Fig. 5a), which is carried with HBV. CCK-8, 5-ethynyl-2-deoxyuridine (EdU) incorporation and colony-forming assays showed that HOIL-1 knockdown prominently inhibited the HepG2.2.15 cell proliferation ability (Fig. 5b–d). In addition, the knockdown of HOIL-1 expression downregulated the percentage of cells in the G1 phase while upregulating cells in S phase (Fig. 5e). The above results indicated that HOIL-1-knockdown inhibited cell proliferation by promoting the S-phase arrest in the cell cycle. To verify the oncogenic role of HOIL-1 in HBV-HCC in vivo, we established a subcutaneous nude mouse model with HOIL-1-silenced HepG2.2.15 cells. We found that the silencing of HOIL-1 significantly alleviated the tumor growth and led to an apparent decrease in tumor weights (Fig. 5f, g). In addition, the IHC staining demonstrated a reduction in the expression of proliferating cell marker Ki-67 (Fig. 5h), which further confirmed the tumor-promoting effect of HOIL-1.

As previously reported, HBV infection confers HCC cells with high metastatic potential and CSC properties^5,27^. Thus, we further investigated whether HOIL-1 played a key role in these processes. We found that HOIL-1 knockdown significantly reduced the expression of mesenchymal markers, including N-cadherin and vimentin, as indicated by western blot (Fig. 5i). The HOIL-1 knockdown inhibited cell migration in vitro and the expression of N-cadherin in vivo (Fig. 5j–l). Furthermore, we found that HOIL-1 silencing significantly impaired tumorsphere formation and reduced CD133^+^ cell population and the proliferative ability of CSCs, and all of these effects were rescued by HOIL-1 overexpression (Fig. 5m–o). Taken together, these results indicated that HOIL-1 possesses oncogenic activities in HBV-HCC.

### HOIL-1 interacts with HBx and promotes its stabilization by reducing its K48 ubiquitination

**Fig. 6:**
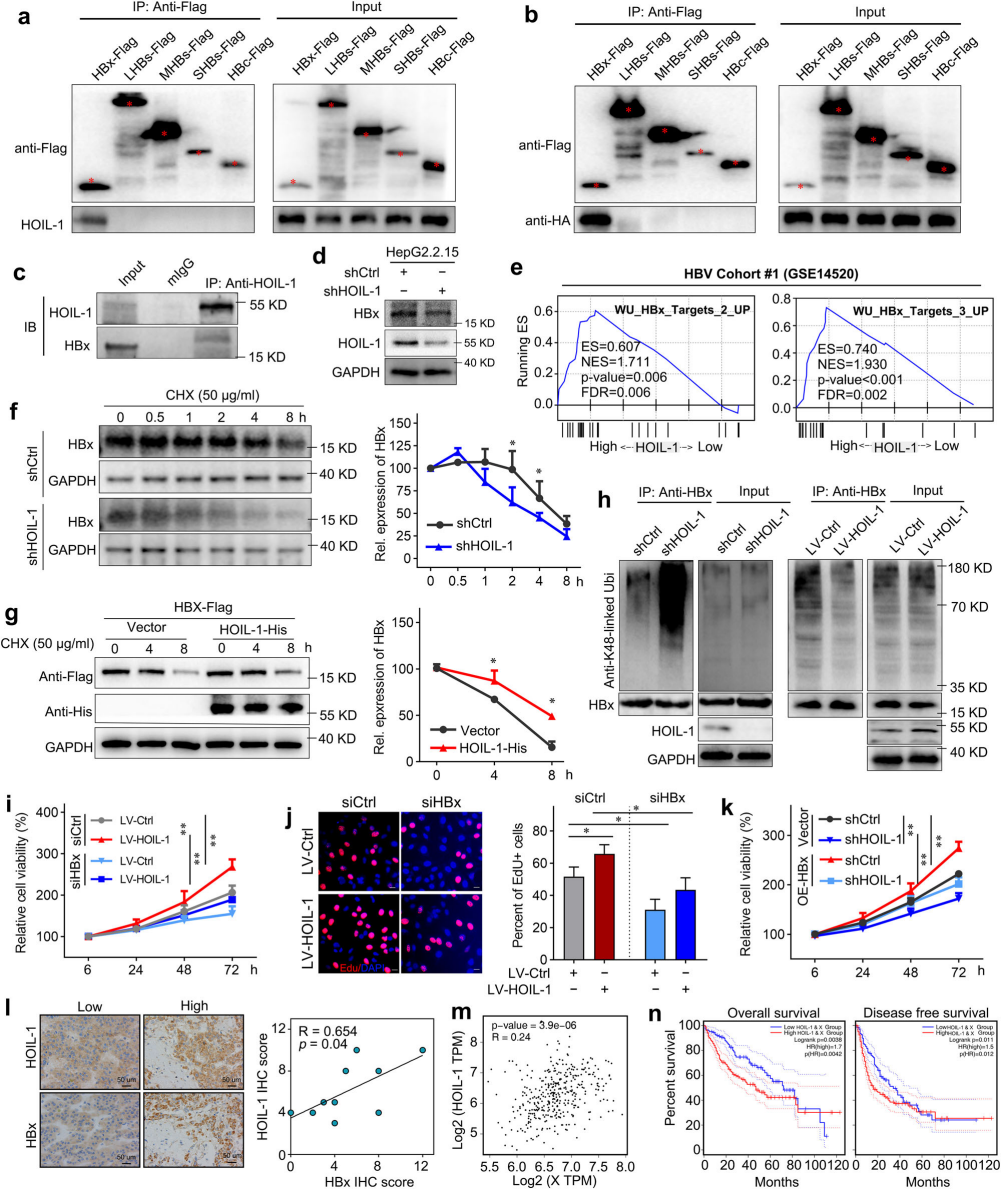
HOIL-1 inhibits the degradation of HBx via inhibiting K48 ubiquitination. **a** The Co-IP and blotting of anti-HOIL-1 or anti-Flag in HepG2 cells transduced with HBx-Flag, LHBs-Flag, MHBs-Flag, SHBs-Flag and HBc-Flag plasmids, respectively. The red star symbol indicates specific bands. **b** The Co-IP and blotting of anti-Flag in HEK293T cells transduced with HOIL-1-HA plasmids combined with HBx-Flag, LHBs-Flag, MHBs-Flag, SHBs-Flag and HBc-Flag plasmids, respectively. The red star symbol indicates specific bands. **c** The Co-IP and blotting of anti-HOIL-1 or anti-HBx in HepG2.2.15 cells. **d** The western blot analysis of indicated proteins in the HOIL-1-knockdown and control cells. **e** The GSEA for HOIL-1 expression in HBx-related gene signatures (WU_HBx_Target_2_UP, WU_HBx_Target_3_UP) in the GSE14520 cohort. **f** The western blot of HBx in HepG2.2.15 cells incubated with cycloheximide at different time points. **g** The western blot of the indicated tag protein detected in HepG2 cells with exogenous HBx-plasmid transfection and incubated with cycloheximide at different time points. **h** The Co-IP for the K48-linked ubiquitination of HBx in the HepG2.2.15 cells after 8 h of MG132 treatment (10 μM). **i**, **j** The effect of the HBx knockdown on the cell proliferation of HepG2.2.15 cells transfected with LV-HOIL-1 or LV-Ctrl detected by CCK-8 (**i**) and EdU incorporation (**j**) assays. **k** The CCK-8 assays detected the effect of HBx overexpression on proliferation of HepG2.2.15 cells. **l** The representative images of IHC staining of HOIL-1 and HBx in HCC tissues and the correlation between HOIL-1 and HBx levels, *n* = 10. **m** The correlation between *HOIL-1* and *HBx* mRNA expression in the TCGA LIHC cohort. **n** The Kaplan–Meier analysis for the OS and DFS of two groups of patients with HCC defined by low and high coexpression of *HOIL-1* and *HBx* in the TCGA LIHC cohort. The Mann–Whitney *U* test was used in **f**, **g** and **i**–**k**, Spearman’s rank correlation test in **l** and the log-rank Mantel–Cox test in **n**. Data are shown as mean ± s.d., *P < 0.05, **P < 0.01. CHX cycloheximide, Co-IP Co-immunoprecipitation assays, IB immunoblot, IF immunofluorescence, IP immunoprecipitated, MG132 N- (benzyloxycarbonyl) leucinylleucinylleucina.

Recent evidence has proven that HBx is paramount in regulating multiple cellular processes in HBV-HCC, including cell proliferation and migration^5,27,28^. Interestingly, we observed an interaction of HOIL-1 with HBx but not with the other HBV-related proteins (HBc, LHBs, MHBs and SHBs) by means of endogenous and exogenous coimmunoprecipitation (Co-IP) assays (Fig. 6a–c). Meanwhile, HOIL-1 knockdown in HepG2.2.15 cells significantly reduced the expression of HBx (Fig. 6d). In addition, GSEA revealed that HBx-related gene sets (WU_HBx_Targets_2_UP and WU_HBx_Targets_3_UP) were strongly enriched in the high HOIL-1 expression group (Fig. 6e). As reported in the previous study, the HBx protein could be rapidly degraded by the proteasome pathway. Because HOIL-1 promoted HCC progression independently of its LUBAC activity, we further investigated the mechanism of how HOIL-1 maintained the stability of HBx. By means of cycloheximide chase assays, we found that HOIL-1 knockdown increased the degradation of HBx, whereas cotransfected HBx-Flag and HOIL-1-His plasmids in HepG2 cells increased the stability of HBx-Flag (Fig. 6f, g). Furthermore, using ubiquitination assays, we noticed that HOIL-1 knockdown increased the K48-linked ubiquitination of HBx, which has been reported to mediate the proteasomal degradation of HBx^10,29^ (Fig. 6h). In addition, we found that HOIL-1 knockdown inhibited the expression of linear ubiquitin chains in HepG2.2.15 cells even under tumor necrosis factor treatment (Supplementary Fig. 11a) but exerted no obvious effect on the M1 ubiquitin levels of HBx (Supplementary Fig. 11b). By analyzing our previous RNA sequence data, we found that HOIL-1 knockdown, which impaired LUBAC formation, inhibited NF-κB pathway activation (Supplementary Fig. 11c), HOIL-1 overexpression showed no notable effects (Supplementary Fig. 11d). In our additional in vitro experiments, we found that HOIL-1 promoted the activation of the NF-κB pathway in HBV-infected HCC cells, which was reversed by HBx knockdown (Supplementary Fig. 11e). Furthermore, HOIL-1 overexpression promoted the MCP-1 expression, which is a targeted gene of NF-κB, and the binding of p65 to the MCP-1 promoter, which were abolished by the HBx knockdown (Supplementary Fig. 12). These findings suggest that HOIL-1 overexpression alone is insufficient to fully activate NF-κB signaling, but it can enhance HBx-mediated NF-κB transcriptional activity. This highlights a potential cooperative interaction between HOIL-1 and HBx in regulating NF-κB activation in HBV-infected cells.

Functionally, by CCK-8 and EdU incorporation assays, we found that HBx knockdown significantly inhibited the proliferation of HepG2.2.15 cells and impaired HOIL-1-mediated protumor activities (Fig. 6i, j). Conversely, HBx overexpression restored the proliferative capacity imparied by HOIL-1 knockdown (Fig. 6k). Clinically, a positive correlation between the HOIL-1 and HBx levels was obtained in our HBV-HCC cohorts (Fig. 6l), which indicated the underlying regulatory relationship between HBx and HOIL-1. Meanwhile, we also observed a positive correlation between the *HOIL-1* and *X* (the gene that encodes HBx protein) mRNA expression in the TCGA cohort (Fig. 6m). In addition, high HOIL-1 and X coexpression was associated with poor prognosis in patients with HCC compared with those with low gene expression (Fig. 6n). Thus, these results suggest that HOIL-1 regulates the stability of the HBx protein by reducing its ubiquitin degradation, thereby promoting the HCC progression.

### HOIL-1 inhibits HBx ubiquitin-mediated degradation by recruiting the deubiquitinating enzyme USP15

**Fig. 7:**
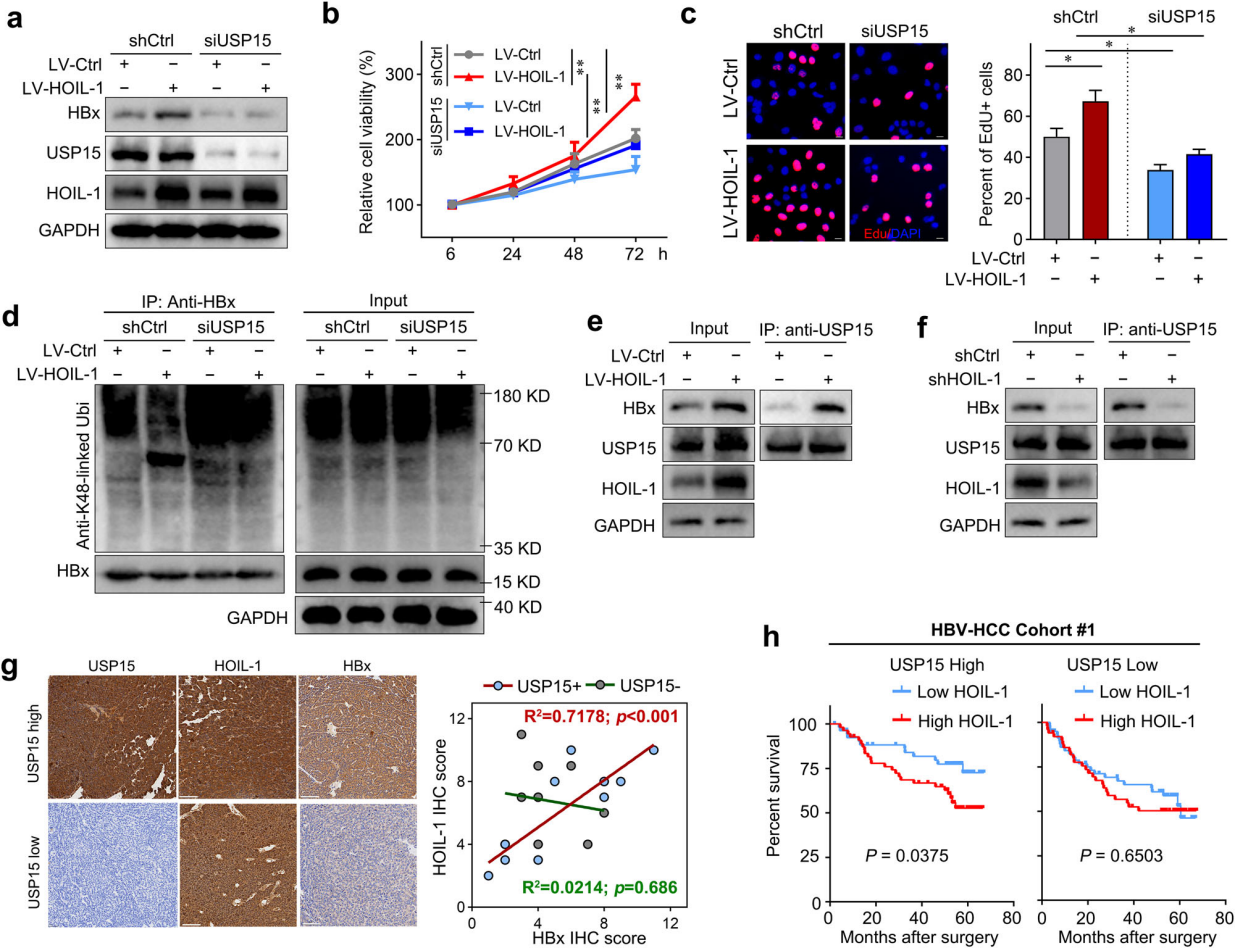
HOIL-1 promotes HBx stabilization via recruiting deubiquitinating enzyme USP15. **a** The western blot of indicated proteins detected in HepG2.2.15 cells. **b** The CCK-8 assays detected the effect of USP15 knockdown on HepG2.2.15 transduced with LV-HOIL-1 or LV-Ctrl. **c** The EdU incorporation detected the effect of USP15 knockdown on HepG2.2.15 transduced with LV-HOIL-1 or LV-Ctrl. **d** The Co-IP for the K48-linked ubiquitination of HBx in the HepG2.2.15 cells after 8 h of MG132 treatment (10 μM). **e** The Co-IP and blotting of anti-HOIL-1 or anti-HBx or USP15 in HepG2.2.15 cells. **f** The Co-IP and blotting of anti-HOIL-1 or anti-HBx or USP15 in HepG2.2.15 cells. **g** The representative images of the IHC staining of HOIL-1, HBx and USP15 in HCC tissues and the correlation between HOIL-1 and HBx levels. **h** The Kaplan–Meier survival analysis of patients in the HBV-HCC cohort (GSE14520) stratified by USP15 and HOIL-1 expression. The patients were divided into USP15-high and USP15-low subgroups and, within each subgroup, further stratified by HOIL-1 expression levels. The Mann–Whitney *U* test was used in **b** and **c**, Spearman’s rank correlation test in **g** and the log-rank Mantel–Cox test in **h**. Data are shown as mean ± s.d., *P < 0.05, **P < 0.01.

**Fig. 8:**
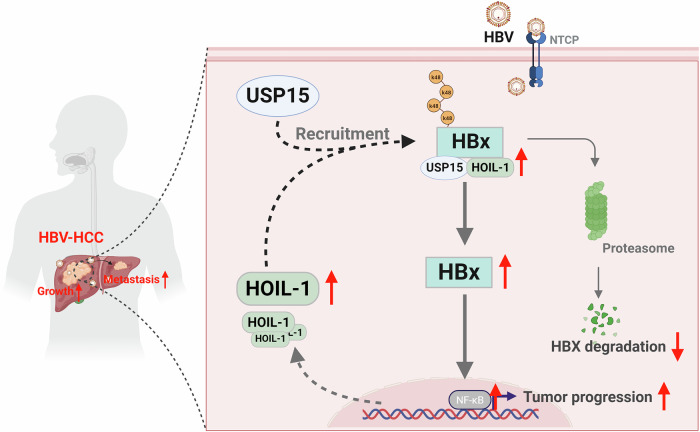
A working model that HOIL-1 promotes the progression of HBV-HCC. The LUBAC component HOIL-1 is upregulated in HBV-HCC; it functions independently of the canonical LUBAC complex. Instead, HOIL-1 recruits the deubiquitinating enzyme USP15 to inhibit the ubiquitin-mediated degradation of HBx, thereby promoting NF-κB signaling activation and contributing to HCC progression.

The deubiquitylating enzyme USP15 has been reported to play a critical role in enhancing HBx stabilization and its transactivation activity^30^. Therefore, we next investigated whether USP15 is involved in HOIL-1-mediated HBx upregulation and its downstream oncogenic effects. Consistently, we found that USP15 knockdown suppressed HBx expression and abolished the upregulation of HBx induced by HOIL-1 overexpression (Fig. 7a). CCK-8 and EdU incorporation assays further showed that USP15 knockdown significantly inhibited the proliferation of HepG2.2.15 cells and attenuated the protumorigenic effects mediated by HOIL-1 (Fig. 7b, c). Moreover, ubiquitination assays revealed that HOIL-1 overexpression reduced the K48-linked ubiquitination of HBx, whereas this effect was abolished by USP15 knockdown (Fig. 7d). Notably, HOIL-1 overexpression enhanced the interaction between HBx and USP15, whereas HOIL-1 knockdown led to a diminished interaction, suggesting that HOIL-1 facilitated USP15-mediated deubiquitination of HBx (Fig. 7e, f). Clinically, we found that HOIL-1 expression was positively correlated with HBx expression in HBV-HCC tissue with high USP15 expression but not in tissues with low USP15 expression (Fig. 7g). Furthermore, the Kaplan–Meier survival analysis revealed that high HOIL-1 expression was associated with poorer prognosis compared with low HOIL-1 expression in the USP15-high subgroup. By contrast, in the USP15-low subgroup, no difference in survival time was observed between patients with high and low HOIL-1 expression (Fig. 7h). Taken together, these results indicate that HOIL-1 promotes HBx stabilization and enhances its oncogenic activity via USP15 (Fig. 8).

## Discussion

In the present Article, we reveal that the components of LUBAC (*HOIL-1*, *HOIP* and *SHARPIN*) and M1-Ubi expression were upregulated in HCC tissues compared with that in the normal control. However, we found that targeting LUBAC activity with the inhibitor HOIPIN-1 did not exert an inhibitory effect on the malignancy of HCC cells, indicating a noncanonical regulation of LUBAC on HCC progression. Furthermore, we found that only the expression of *HOIL-1* but not *HOIP* or *SHARPIN* was elevated in HBV-HCC tissues and related to poor prognosis in HBV-HCC cohorts. Functionally, targeting HOIL-1 significantly inhibited the growth and metastasis of HBV-HCC cells in vitro and in vivo. Mechanistically, HOIL-1 interacted with HBx and inhibited its K48-linked ubiquitination by recruiting the deubiquitinating enzyme USP15, thereby reducing its degradation. Thus, our study proposed that HOIL-1 promotes the stability of HBx by inhibiting the K48 ubiquitination of HBx and is a potential target for HBV-HCC therapeutics.

About 700 RING finger family proteins have been identified, most of which contain the RING-in-between-RING (RBR) domain. Unlike most E3 ubiquitin ligases that mediate the proteolytic polyubiquitination, recent studies revealed that several RING family ubiquitin ligases promoted atypical ubiquitination on their substrates. For example, HOIL-1 could cooperate with HOIP and SHARPIN to form LUBAC to mediate the linear ubiquitination of IKKγ and promote the activation of NF-κB signaling^31^. In previous studies, it had been reported that the expression of HOIL-1 and SHARPIN was upregulated in tumor tissues^22,32^ and exerted oncogenic functions by stabilizing the expression HOIP and promoting the transactivation of Versican expression; however, it was independent of their M1-Ubi activity. LUBAC-mediated M1-Ubi has been gradually studied since it was first found to be a new type of ubiquitination^31^ and affected the progression of multiple cancers^18,33,34^. However, its role in HCC development and progression is poorly understood. The present study revealed that LUBAC-mediated M1-Ubi and LUBAC components were significantly elevated in HCC tissues and correlated with poor prognosis. However, its function does not depend on LUBAC activity. It has been reported that HBV is a primary etiology of chronic viral hepatitis and further leads to the occurrence of HCC. We performed analyses with the public and our cohorts to further explore the role of LUBAC components in HBV-HCC. However, we found that only *HOIL-1*, rather than *HOIP* or *SHARPIN*, was upregulated in the HBV-HCC compared with the non-HBV-HCC tissues, indicating the specific interaction between HOIL-1 and the HBV components. In further exploration, the upregulated expression of HOIL-1 was associated with high AFP levels, large tumor size and poor prognosis in both public and our HBV-HCC cohorts. Although high HOIL-1 expression appeared to be beneficial for long-term OS beyond approximately 100 weeks and showed no difference from low HOIL-1 expression in disease-free survival in the TCGA LIHC cohort, we believe this phenomenon may be due to the insufficient number of patients beyond the 100-week cutoff. Notably, the results from the GEO dataset and our cohort were consistent. Moreover, inhibiting HOIL-1 expression alleviated the growth and metastasis of HBV-carried HCC cells in vitro and in vivo.

HOIL-1, the component of LUBAC, is a 58 kDa protein that contains the N-terminal ubiquitin-like domain, the Npl4 zinc finger (NZF) domain and the catalytic carbon terminal RBR domain^35^. Recently, serveral E3 ubiquitin ligases have exhibited abnormal expression in tumors, making them valuable diagnostic markers and drug targets^36^. Previous studies have revealed that publicly available databases and in vitro analysis have determined that the mRNA expression of *HOIL-1* in HCC cells and tissues was significantly higher than those of normal counterparts^22,23^. For factors upregulating the expression of HOIL-1, Queisser et al. considered that the hypoxia-inducible factor regulated the expression of HOIL-1 in breast cancer^33^. In addition, HOIL-1 was identified as a copy number variation-driven gene by screening out copy number variation-driven differentially expressed genes in liver cancer^37^. In the present Article, we demonstrate that HOIL-1 expression was upregulated in HBV-HCC tissues compared with that in non-HBV-HCC tissues, indicating a positive regulation of HBV on the expression of HOIL-1. Numerous gene expressions were regulated by HBV infection via various mechanisms, including the HBx pathway^38^, *N*^6^-methyladenosine modification^39^ and DNA methylation^40^. Increasing evidence indicates that HBx, the transactivating factor of viral genes, is mainly involved in regulating HCC occurrence, proliferation, invasion, migration and glucose metabolism. HBx interacts with cellular proteins to activate various signaling pathways or affect gene expression by epigenetic regulation. To further explore the mechanism of HOIL-1 in HBV-HCC, we analyzed whether HOIL-1 interacted with HBV-associated protein, including HBx, HBc, LHBs, MHBs and SHBs, and found that HOIL-1 only interacted with HBx. Furthermore, HOIL-1 regulated the stability of the HBx protein through recruiting USP15 to reduce HBx K48 ubiquitination, thereby promoting HBV-HCC progression. Importantly, our findings revealed a previously unrecognized interaction between HOIL-1 and HBx that was essential for downstream oncogenic signaling in HBV-related hepatocarcinogenesis. Given the technical challenges associated with directly inhibiting HOIL-1’s enzymatic activity—due to its role as part of a larger E3 ligase complex—we proposed that targeting the HOIL-1–HBx interaction may represent a more tractable therapeutic strategy. Structure-based drug design or peptide-mimetic approaches aimed at disrupting this protein–protein interface could offer a novel avenue for intervention. Furthermore, modulating adaptor proteins or signaling nodes downstream of HOIL-1 also provided alternative druggable targets worthy of future investigation.

## Supplementary information

Supplementary Information
