# Supplementary material for: The LUBAC subunit HOIL-1 promotes the progression of HBV-associated hepatocellular carcinoma independently of linear ubiquitination
Source: Exp Mol Med. 2025 Oct 6;57(10):2317–30. doi: 10.1038/s12276-025-01556-4 (PMC12586429; doi:10.1038/s12276-025-01556-4)
Supplement: Supplementary file 1 — Supplementary Information [file 12276_2025_1556_MOESM1_ESM.pdf]

The LUBAC subunit HOIL-1 promotes the progression of HBV-associated hepatocellular carcinoma independently of linear ubiquitination

Tables of contents

Supplementary Table 1..2

Supplementary Table 2..3

Supplementary Fig. 1..4

Supplementary Fig. 2..5

Supplementary Fig. 3..6

Supplementary Fig. 4..7

Supplementary Fig. 5..8

Supplementary Fig. 6..9

Supplementary Fig. 7..10

Supplementary Fig. 8..11

Supplementary Fig. 9..12

Supplementary Fig. 10..13

Supplementary Fig. 11..14

Supplementary Fig. 12..16

Supplementary Table 1. Clinical Characteristics of 147 HCC Patients

Patient characteristics	Number
No. of patients	147
Age, y, (≤50/ >50), n	74/73
Sex, male/female, n	129/18
HbsAg, negative/ positive, n	20/127
ALT, (≤40 *U/L*/>40 *U/L*), n	77/68
AST, (≤40 *U/L*/>40 *U/L*), n	57/90
AFP (≤25 ng/mL/≥25ng/mL), n	45/102
tumour size (≤5 cm/>5 cm), n	70/77
tumor multiplicity, solitary/ multiple, n	115/32
tumor thrombus, no/ yes, n	127/20
tumor differentiation, I+II/III+IV, n	133/14

Supplementary Table 2: The RNA sequencing coverage and quality statistics. RNA quality was assessed on an Agilent 2100 Bioanalyzer (Agilent Technologies, Palo Alto, CA, USA) and checked using RNase free agarose gelelectrophoresis. After total RNA was extracted, eukaryotic mRNA was enriched by Oligo(dT) beads. Then the enriched mRNA was fragmented into short fragments using fragmentation buffer and reversly transcribed into cDNA by using NEBNext Ultra RNA Library Prep Kit for Illumina (NEB #7530, New England Biolabs, Ipswich, MA, USA). The purified double-stranded cDNA fragments were end repaired, A base added, and ligated to Illumina sequencing adapters. The ligation reaction was purified with the AMPure XP Beads (1.0X). And polymerase chain reaction (PCR) amplified. The resulting cDNA library was sequenced using Illumina Novaseq6000 by Gene Denovo Biotechnology Co. (Guangzhou, China). The Raw data were uploaded to the Genome Sequence Archive (GSA) database (GSA accession No. HRA002273).

Sample Name	Total number of sequenced reads	Total number of uniquely mapped reads	RNA integrity number (RIN)	Ratio of all reads aligned to rRNA regions to total uniquely mapped reads (rRNA rate)	Ratio of exon-mapped reads to total uniquely mapped reads (Expression Profile Efficiency)	Total number of detected transcripts with reads≥1
HCCLM3_LvCtrl_1	47633608	43893032	≥6	92.15	84.92	20323
HCCLM3_LvCtrl_2	43798786	40405765	≥6	92.25	84.82	20323
HCCLM3_LvCtrl_3	50754894	46997872	≥6	92.60	84.59	20323
HCCLM3_LvHOIL1_1	52199954	46546178	≥6	90.91	82.13	20323
HCCLM3_LvHOIL1_2	54324968	49141041	≥6	90.46	82.58	20323
HCCLM3_LvHOIL1_3	42373484	38183097	≥6	90.11	82.31	20323
Huh7_shCtrl_1	47287638	44268614	≥6	93.62	87.64	20323
Huh7_shCtrl_2	42552464	39628287	≥6	93.13	87.58	20323
Huh7_shCtrl_3	42721552	39760219	≥6z	93.07	87.37	20323
Huh7_shHOIL1_1	35885182	33388198	≥6	93.04	86.63	20323
Huh7_shHOIL1_2	42330260	39196405	≥6	92.60	86.39	20323
Huh7_shHOIL1_3	51856830	48437409	≥6	93.41	86.55	20323

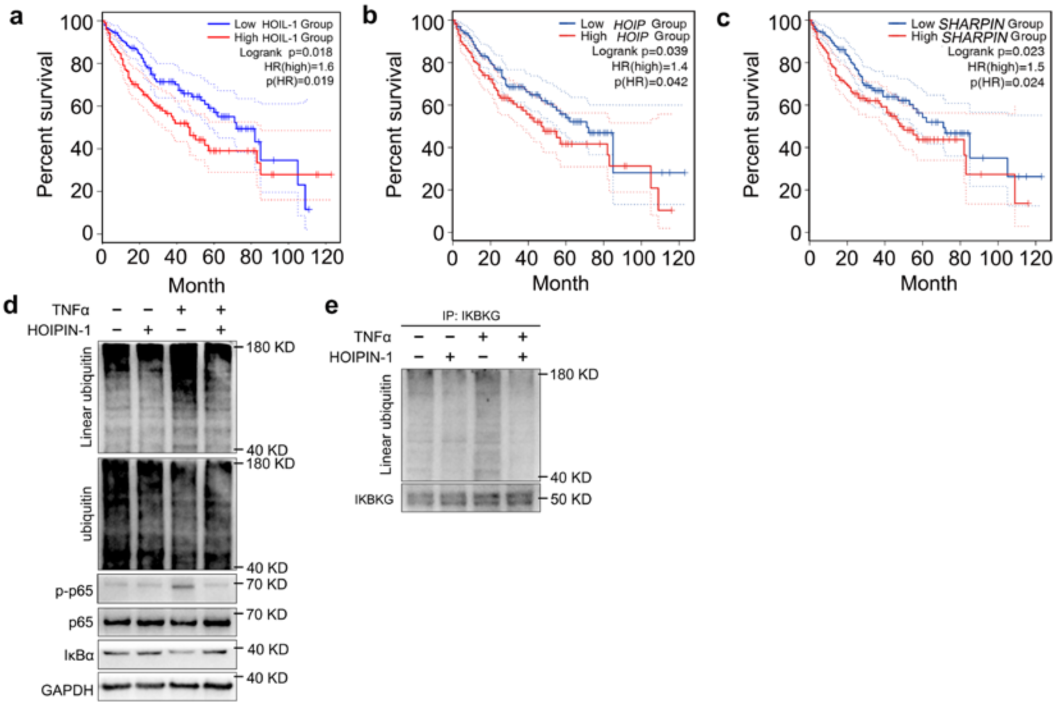

Supplementary Fig. 1. The expression and activity of LUBAC in HCC. (a-c) The association between the expression of LUBAC components (HOIL-1, HOIP, SHARPIN) and HCC patients' overall survival time in TCGA LIHC cohort. (d) Western blot of the expression of linear ubiquitin, ubiquitin, p-p65, p65 and IκBα in Huh7 cells under 20 ng/ml TNFα treatment for 0.5h. (e) Western blot of linear ubiquitin levels of IKBKG. (a-c) log-rank Mantel-Cox test. LIHC, liver hepatocellular carcinoma; TCGA, The Cancer Genome Atlas.

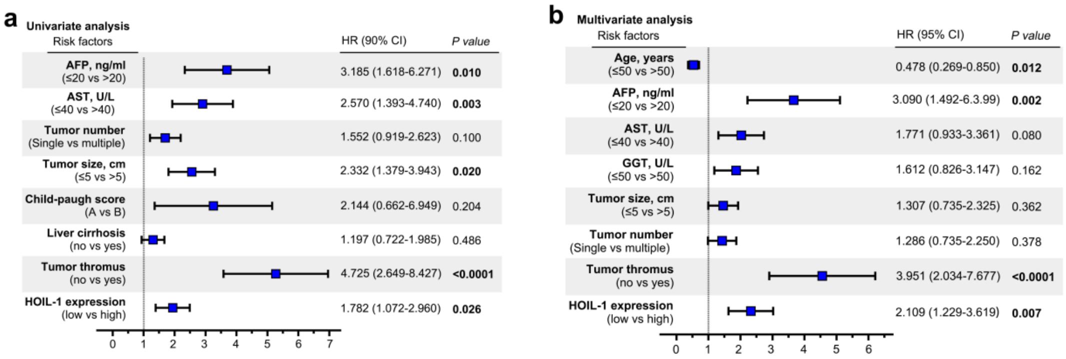

Supplementary Fig. 2. Univariate (a) and multivariate (b) regression analyses of association of the HOIL-1 expression and clinicopathological parameters with HCC patients' overall survival time in our cohort.

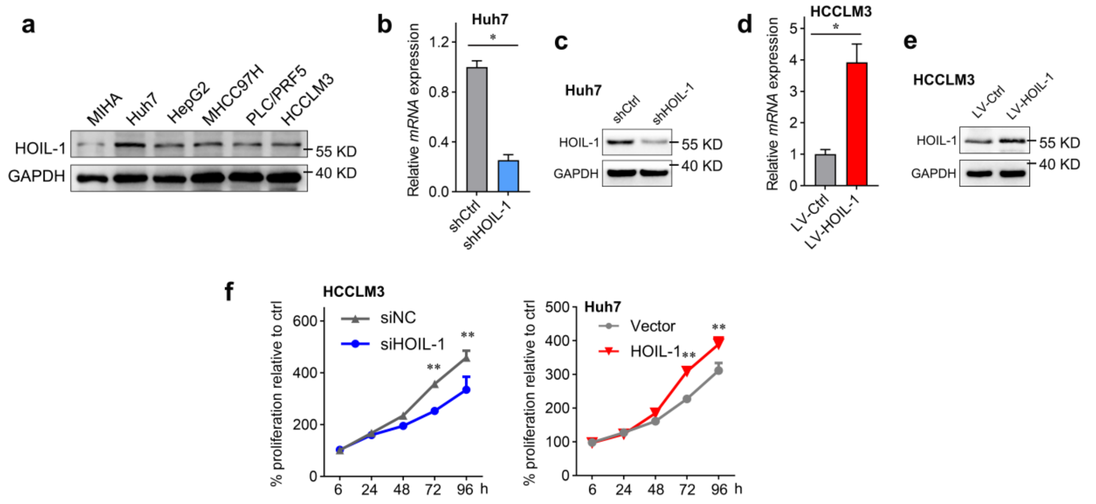

Supplementary Fig. 3. The expression of HOIL-1 in HCC cells. (a) The basal expression of HOIL-1 in HCC cell lines and normal hepatocytes. (b) The mRNA expression levels of HOIL-1 in Huh7 cells transfected with shHOIL-1 or shCtrl. (c) The protein expression levels of HOIL-1 in Huh7 cells transfected with shHOIL-1 or shCtrl. (d) The mRNA expression levels of HOIL-1 in HCCLM3 cells transfected with LV-HOIL-1 or LV-Ctrl. (e) The protein expression of HOIL-1 in HCCLM3 cells transfected with LV-HOIL-1 or LV-Ctrl. (f) CCK8 assays detected the proliferation of HCC cells.. Data were shown as Mean ± SD, *p <0.05, **p <0.01; (b, d, f) Mann-Whitney U test.

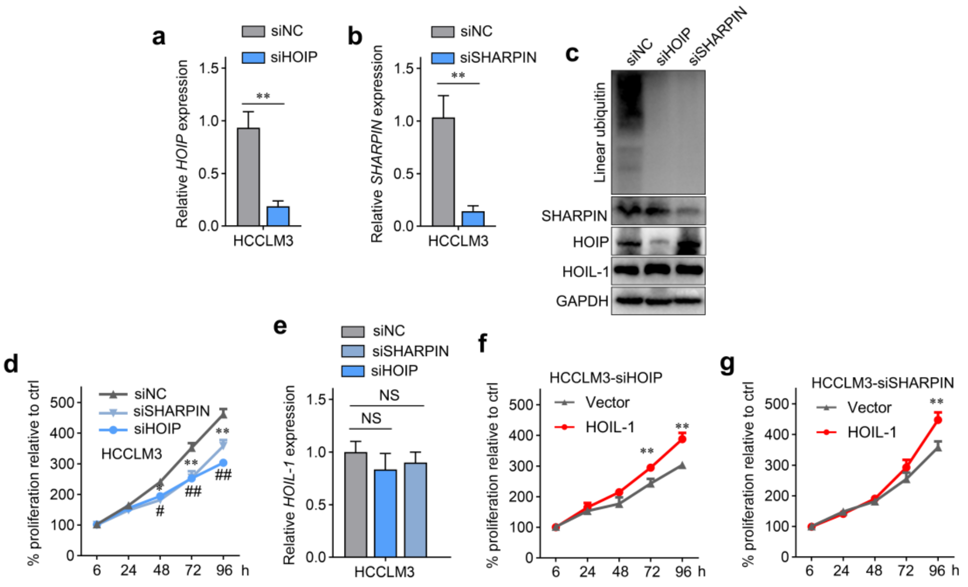

Supplementary Fig. 4. HOIL-1 promotes HCC cells proliferation independently of LUBAC. (a) RT-qPCR analysis of HOIP expression in HCCLM3 cells transfected with siHOIP. (b) RT-qPCR detected the SHARPIN expression in HCCLM3 cells transfected with siSHARPIN. (c) Western blot detected the expression of HOIL-1, SHARPIN, HOIP and linear ubiquitin in HCCLM3 cells. (d) CCK8 assays assesing the proliferation of HCCLM3 cells. (e) RT-qPCR analysis the HOIL-1 expression. (f-g) The effects of HOIL-1 overexpression on the proliferation capacity of HCCLM3 cells with HOIP or SHARPIN knockdown. Data were shown as Mean ± SD, NS, not significant, **p <0.01; (a, b, d-g) Mann-Whitney U test.

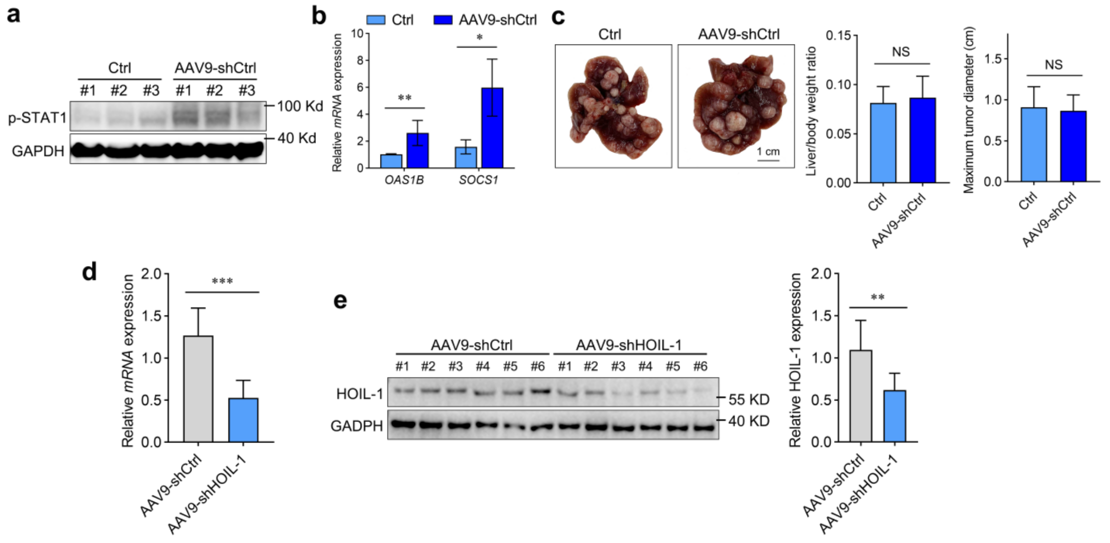

Supplementary Fig. 5. The impact of AAV9-shHOIL-1 on HCC *in vivo*. (a-b) The expression of p-STAT1, OAS1B, SOCS1 in liver of mice treated with AAV9-shCtrl or Ctrl. (c) The gross images of liver, and the quantification of liver/body weight ratio and maximum tumor diameter. (d) The mRNA expression levels of HOIL-1 in DEN/CCl_4-induced mouse model. (e) The protein expression levels of HOIL-1 in DEN/CCl_4-induced mouse model, and the quantification. Data were shown as Mean ± SD, NS, not significant, *p <0.05, **p <0.01, ***p <0.001; (b-e) Mann-Whitney U test.

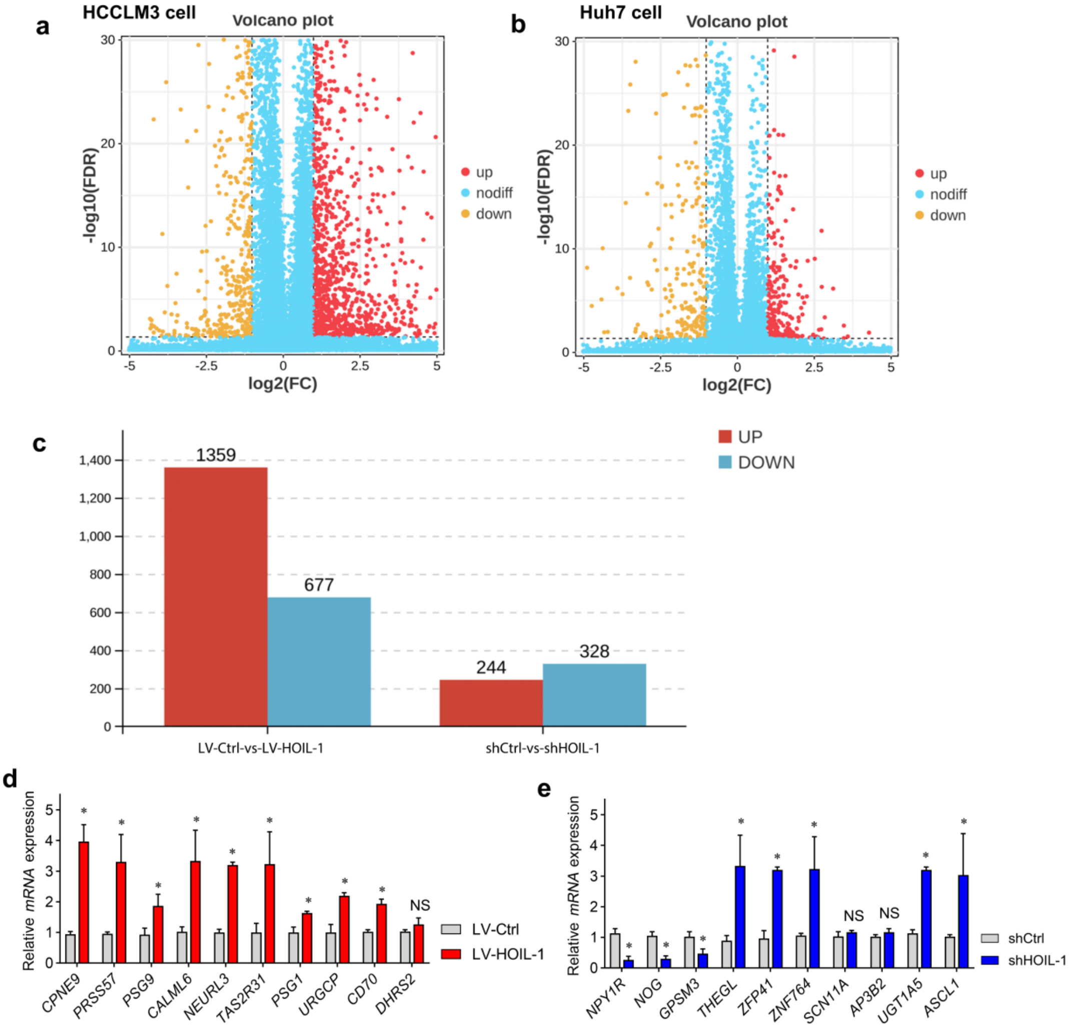

Supplementary Fig. 6. RNA-Seq of the gene expression in HCCLM3 and Huh7 cell. (a) Volcano plot of gene expression of HCCLM3 cells treated as indicated. (b) Volcano plot of gene expression of Huh7 cells treated as indicated. (c) The bar plot of the number of up-regulated and down-regulated genes in HCCLM3 and Huh7 cells treated as indicated. (d-e) RT-qPCR was performed to detect the top 10 differentially expressed genes following HOIL-1 knockdown in Huh7 and overexpression in HCCLM3 cells. Data were shown as Mean ± SD, NS, not significant, *p <0.05; (d-e) Mann-Whitney U test.

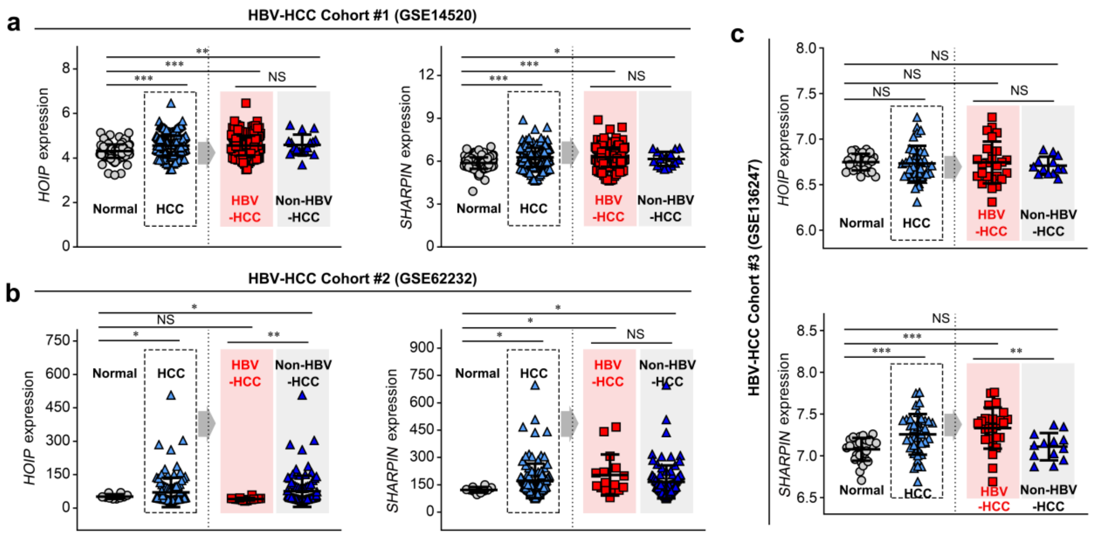

Supplementary Fig. 7. The expression of *HOIP* and *SHARPIN* do not correlate with tumor progression in HBV-associated HCC. (a-c) The expression of *HOIP* and *SHARPIN* in GSE14520 (a), GSE62232 (b), and GSE136247 (c) cohort. Data were shown as Mean ± SD, *p <0.05, **p <0.01, ***p <0.001, NS, not significant; (a-c) Kruskal-Wallis H test.

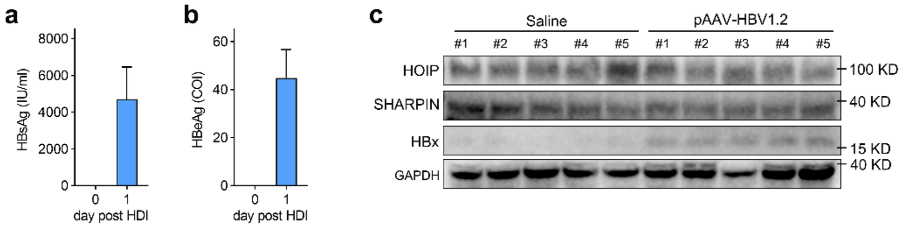

Supplementary Fig. 8. Characteristic of pAAV-HBV1.2 mouse models. (a-b) The serum HBsAg (a) and HBeAg (b) levels in mice detected by Elisa at the 24h post hydrodynamic injection. (c) Western blot of HOIP, SHARPIN, and HBx in mice liver at the 24h post hydrodynamic injection.

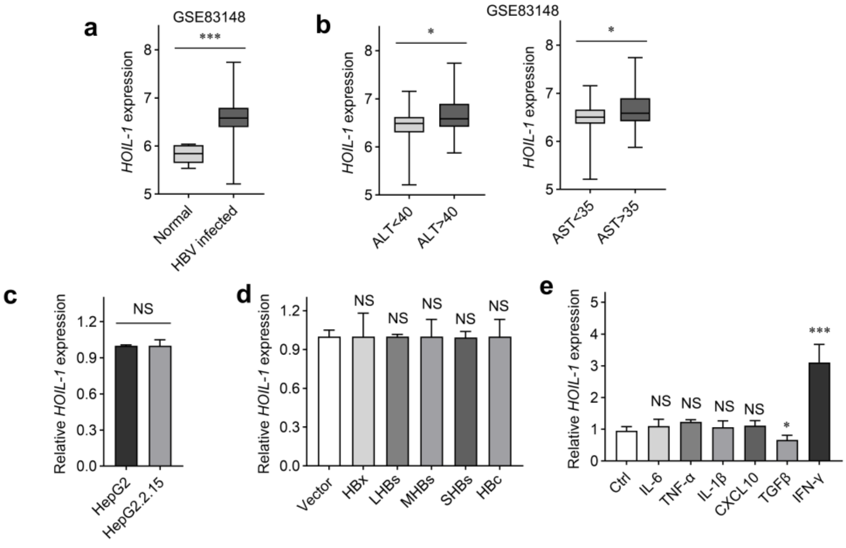

Supplementary Fig. 9. The impact of HBV infection on HOIL-1 expression. (a) The HOIL-1 expression in liver tissues from HBV infected individuals and healthy controls in GSE83148 cohorts. (b) The HOIL-1 expression in liver tissues from HBV infected individuals with elevated alanine aminotransferase (ALT) or aspartate aminotransferase (AST) levels compared to their controls. (c) The HOIL-1 expression in HepG2 and HepG2.2.15 cells. (d) The HOIL-1 expression in HepG2 cell transfected with HBV-related plasmids. (e) The effect of cytokines on HOIL-1 expression in HepG2.2.15 cells. Data were shown as Mean ± SD, *p <0.05, ***p <0.001, NS, not significant; (a-e) Mann-Whitney U test.

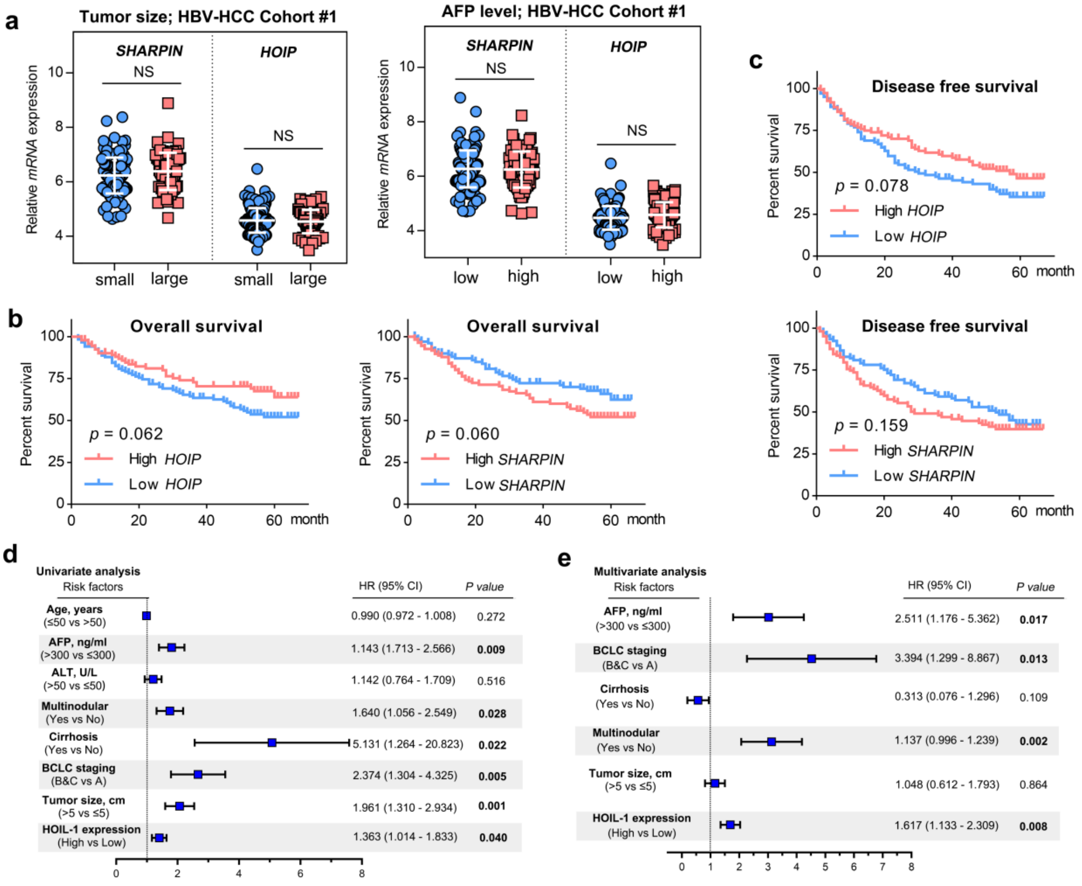

Supplementary Fig. 10. The association of HOIL-1 expression with patients' prognosis in HBV-HCC cohort. (a) The association between *HOIP* and *SHARPIN* expression and tumor size and AFP level. (b-c) The association between *HOIP* and *SHARPIN* expression and HCC patients' overall survival time and disease-free survival time in HBV-HCC cohort #1 (GSE14520). (d-e) Univariate (d) and multivariate (e) regression analyses of association of the HOIL-1 expression and clinicopathological parameters with HCC patients' overall survival time in HBV-HCC cohort #1 (GSE14520). Data were shown as Mean ± SD, NS, not significant; (a) Mann-Whitney U test. (b, c) log-rank Mantel-Cox test.

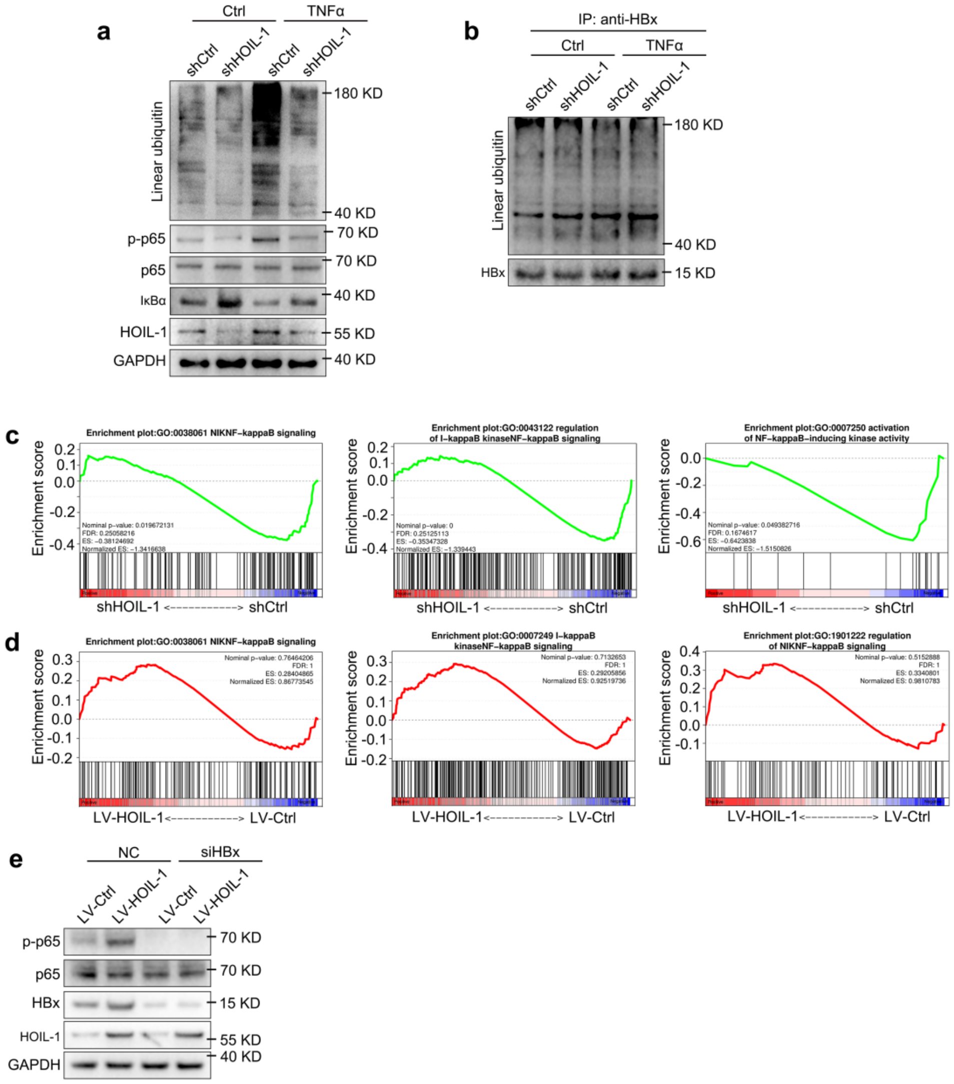

Supplementary Fig. 11. The effects of HOIL-1 knockdown on the expression of linear ubiquitin chains and HBx M1 ubiquitin. (a) Western blot of the expression of linear ubiquitin, ubiquitin, p65, p-p65,IκBα and HOIL-1 in HepG2.2.15 cell under 20 ng/ml TNFα treatment for 0.5 hour. (b) Western blot of the expression of HBx M1 ubiquitin in HepG2.2.15 cell under 20 ng/ml TNFα treatment for 0.5 hour. (c, d) GSEA for NFκB signaling related gene signatures in HOIL-1 knockdown (c) and

overexpressed (d) HCC cells. (e) Western blot of the expression of p65, p-p65, HBx, and HOIL-1 in HepG2.2.15 cell.

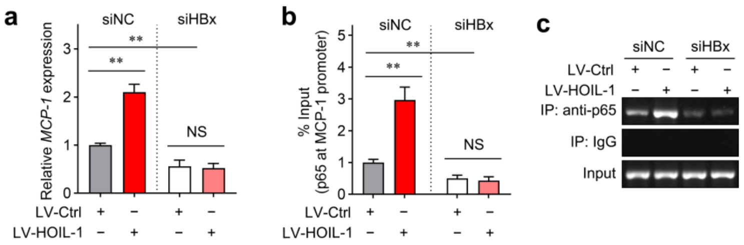

Supplementary Fig. 12. HOIL-1 promotes NFκB signaling activation dependent on HBx in HBV-infected HCC. (a) RT-qPCR analysis MCP-1 expression in HepG2.2.15 cells. (b) ChIP-qPCR analysis of p65 enrichment at the MCP-1 promoter region in HepG2.2.15 cells under indicated treatment. Chromatin was immunoprecipitated using anti-p65 antibody, and enrichment was quantified by qPCR using primers specific for the MCP-1 promoter. (c) Ascertainment of p65 binding to the MCP-1 promoter region using ChIP-PCR. Data were shown as Mean ± SD, **p <0.01, NS, not significant; (a-b) Mann-Whitney U test.
